# Mapping the mHealth Nexus: A Semantic Analysis of mHealth Scholars’ Research Propensities Following an Interdisciplinary Training Institute

**DOI:** 10.3390/app15116252

**Published:** 2025-06-02

**Authors:** Junpeng Ren, Jinwen Luo, Yingshi Huang, Vivek Shetty, Minjeong Jeon

**Affiliations:** 1 Department of Statistics and Data Science, University of California, Los Angeles (UCLA), Los Angeles, CA 90095, USA; 2 Department of Education, University of California, Los Angeles (UCLA), Los Angeles, CA 90095, USA; 3 Division of Diagnostic and Surgical Sciences, School of Dentistry, University of California, Los Angeles (UCLA), Los Angeles, CA 90095, USA

**Keywords:** interdisciplinary research, publication analysis, research trajectories, topic identification, visualization

## Abstract

Interdisciplinary research catalyzes innovation in mobile health (mHealth) by converging medical, technological, and social science expertise, driving critical advancements in this multifaceted field. Our longitudinal analysis evaluates how the NIH mHealth Training Institute (mHTI) program stimulates changes in research trajectories through a computational examination of 16,580 publications from 176 scholars (2015–2022 cohorts). We develop a hybrid analytical framework combining large language model (LLM) embeddings, Uniform Manifold Approximation and Projection (UMAP) dimensionality reduction, and Hierarchical Density-Based Spatial Clustering of Applications with Noise (HDBSCAN) clustering to construct a semantic research landscape containing 329 micro-topics aggregated into 14 domains. GPT-4o-assisted labeling identified mHealth-related publications occupying central positions in the semantic space, functioning as conceptual bridges between disciplinary clusters such as clinical medicine, public health, and technological innovation. Kernel density estimation of research migration patterns revealed 63.8% of scholars visibly shifted their publication focus toward mHealth-dense regions within three years post-training. The reorientation demonstrates mHTI’s effectiveness in fostering interdisciplinary intellect with sustained engagement, evidenced by growth in mHealth-aligned publications from the mHTI scholars. Our methodology advances science of team science research by demonstrating how LLM-enhanced topic modeling coupled with spatial probability analysis can track knowledge evolution in interdisciplinary fields. The findings provide empirical validation for structured training programs’ capacity to stimulate convergent research, while offering a scalable framework for evaluating inter/transdisciplinary initiatives. The dual contribution bridges methodological innovation in natural language processing with practical insights for cultivating next-generation mHealth scholarship.

## 1. Introduction

Interdisciplinary research has been empirically demonstrated to drive innovation through the synthesis of diverse perspectives and methodologies to address complex healthcare challenges [1–5]. The integrative approach has attracted significant attention from science of science and team science domains, which investigate both knowledge production dynamics and the evolution of scientific collaborations [6–8]. The mHealth Training Institute (mHTI) represents a strategic implementation of these principles [9], creating an ecosystem where interdisciplinary collaboration in mobile health (mHealth) flourishes through structured convergence of medical, technological, and social science expertise.

As an NIH-funded initiative, mHTI annually recruits 30 scholars from diverse disciplines to collaboratively address pressing healthcare challenges through intensive team-based projects. The program’s unique structure combines webinars, sandboxes, and in-person collaborations to facilitate knowledge integration across disciplines. The mHTI serves as a unique observatory for analyzing interdisciplinary collaboration dynamics and knowledge evolution across three dimensions: formation mechanisms, topic emergence, and intellectual diffusion patterns. Here, we focus on the last two dimensions.

This study is driven by a set of interconnected research questions designed to unravel the complexities and the migration of research topics within the mHealth domain. At its core, this investigation begins by addressing a foundational question (Q1): What research topics have the mHTI scholars published on? Identifying the thematic areas explored by researchers is crucial for mapping the intellectual landscape and laying the foundation for further knowledge discovery. Building on this foundation, this study delves into a second critical question (Q2): Of all the articles published by the mHTI scholars, which are related to mHealth topics and how? Addressing this question is essential for evaluating how effectively mHTI sustains scholarly engagement with mHealth—a core mandate of the training initiative. However, identifying mHTI-aligned publications remains challenging: mHealth’s interdisciplinary nature creates semantic ambiguity (e.g., overlaps with telemedicine, digital health, or AI diagnostics), while automated keyword or NLP methods risk misclassifying works at interdisciplinary intersections or niche subdomains (e.g., sensor-driven behavioral interventions versus generic wearable tech studies). Finally, based on the findings of Q1 and Q2, we explore a more challenging question (Q3): How have the research interests of the participating mHTI scholars changed over time? To this end, we track scholars’ research topic interests shifts and investigate whether scholars align their research interests more closely with mHealth field after participating in the mHTI program.

The remainder of this paper is structured as follows: In Section 2, we provide some background and review on relevant topics. In Section 3, we describe the methods we adopt to answer the above research questions. We then present the data analysis results and findings in Section 4. We conclude our paper in Section 5 with a summary and discussions on limitations and future research directions.

## 2. Background and Review

### 2.1. Interdisciplinary Research and mHealth

Interdisciplinary research is characterized by the integration of knowledge and methods from multiple disciplines to address complex problems [10]. Menken and Keestra [1] describe practical ways to integrate disciplinary perspectives, while Okamura et al. [2] show that publications integrating multiple fields earn greater impact measured by citations than single-discipline work. Large-scale studies reinforce these findings: Fortunato et al. [6] link cross-field collaboration to many scientific advances; Porter and Rafols [7] trace a steady rise in collaborations between neighboring disciplines; and Wuchty, Jones, and Uzzi [8] report the growing dominance of team-based research, showing that collaborative efforts often yield higher-impact outputs. These patterns are directly relevant to mobile health (mHealth), which sits at the crossroads of medicine, public health, psychology, computing, and engineering.

Mobile health is a prime example of an interdisciplinary field, combining medical expertise, technological innovation, and social science insights to develop solutions for healthcare delivery and patient management [3]. Garai et al. [5] list practical design requirements—such as sustained user engagement and context-appropriate interfaces—showing that attention to these cross-disciplinary details is essential for lasting impact. Iyawa et al. [3] extend this idea with a framework that links technical, clinical, and organizational elements into a single “innovation ecosystem”. Looking back over the field’s development, Istepanian [4] traces mHealth’s shift toward broader digital-health approaches that depend on diverse expertise.

Work in mHealth covers a wide range of applications from remote monitoring to mental-health support and therefore demands collaboration across disciplinary boundaries [3,4,11,12]. Silva et al. [11] review how mobile tools already improve access to care, while Steinhubl et al. [12] outline how faster connectivity and greater computing power are changing clinical research and everyday practice. Together, these studies demonstrated a key point: successful mHealth solutions emerge when medical expertise, technological design, and social science perspectives work together.

While integrating interdisciplinary views offers significant advantages, such as fostering innovation and generating novel insights, it also presents challenges, including communication barriers and the need for specialized training [13,14]. To mitigate these barriers, structured training programs, such as the mHTI [15], play a critical role in fostering interdisciplinary collaboration by providing researchers with the skills and networks needed to navigate cross-disciplinary challenges [9]. These programs not only facilitate knowledge exchange but also help build the research trajectories of participating scholars, encouraging them to explore new methodologies and perspectives. However, the long-term impact of such programs on scholars’ research interests and productivity remains underexplored, highlighting a gap in the literature that this study seeks to address.

### 2.2. Topic Modeling in Scientific Research

Topic modeling has emerged as a powerful tool for analyzing large corpora of scientific literature, enabling researchers to identify latent themes and trends within text data. Techniques such as Latent Dirichlet Allocation (LDA) [16] and Latent Semantic Indexing (LSI) [17] have been widely applied to uncover research topics. For example, an LDA-based topic model can successfully cluster a large collection of scientific publications by research discipline [18]; and both LDA and LSI haven been applied to a corpus of interdisciplinary journal articles and found the latent topics corresponded to major academic fields [19]. These approaches highlight the potential of topic modeling to map the intellectual landscape of scientific fields and identify emerging areas of interest.

Recent advancements in topic modeling, such as BERTopic [20] and TopicGPT [21], have further enhanced the interpretability and adaptability of these methods. BERTopic leverages transformer-based language models to generate coherent topic representations, while TopicGPT uses large language models (LLMs) to produce natural language labels and descriptions for topics, aligning closely with human categorizations. These innovations address limitations of traditional topic models, such as the ambiguity of bag-of-words representations, and provide more intuitive and dynamic ways to explore scientific literature.

### 2.3. Applications of Topic Modeling in mHealth and Digital Health

Topic modeling has been increasingly applied to analyze research trends in mHealth and digital health. For example, bibliometric analyses have used topic modeling to identify key themes and collaboration patterns in mHealth research [22], revealing a growing emphasis on topics such as telehealth, wearable devices, and AI-driven interventions. Similarly, studies have employed topic modeling to explore public perceptions of mental health technologies. For example, LDA was applied to user reviews of AI mental health apps, revealing dominant themes such as emotional support and therapeutic alternatives [23]. Ahmed et al. [24] analyzed over 200,000 chatbot app reviews and identified recurring themes of empathy, usability, and privacy concerns that shape trust. Digital health literature trends have been investigated using BERTopic and identified emerging topics related to behavioral support and remote intervention strategies [22]. These applications demonstrate the utility of topic modeling in uncovering the semantic structure of medical and health-related text data, as well as identifying emerging trends in interdisciplinary mobile health research.

### 2.4. Research Gaps and Contributions

Existing evaluation approaches primarily rely on comparisons of pre- and post-training survey responses, which are often subjective and limited in scope [25]. This narrow focus on outcome variables makes it difficult to capture sustained or structural changes in scholars’ academic trajectories. To address this limitation, some studies have adopted more objective metrics, such as publication records and grant acquisition. It was found that participation in a clinical and translational research Master of Science degree program was associated with significantly higher post-training publication output, using publication counts as a measure of training effectiveness [26]. Knapke et al. [27] reported that graduates of the same program were substantially more likely to receive NIH funding than non-participants, with grant awards serving as another key metric. These findings highlight the value of research outputs in assessing long-term program effectiveness. However, such studies often focus narrowly on quantitative measures like publication counts while overlooking how training programs may influence scholars’ research interests and collaborative patterns. This oversight is particularly relevant for interdisciplinary research training programs such as mHTI, where shifts in research focus and collaboration dynamics are critical outcomes. Regarding research specifically focused on the mHTI program, Ho et al. [9] employed Separable Temporal Exponential Random Graph Models (STERGMs) to model the intra-program communication networks of three annual cohorts of mHTI participants from 2017 to 2019, providing recommendations to enhance interdisciplinary scholar interactions. However, such analyses primarily focus on collaboration dynamics during the short-term training program, rather than examining how the program influences scholars’ long-term research interests beyond their immediate networks.

**Figure 1.**
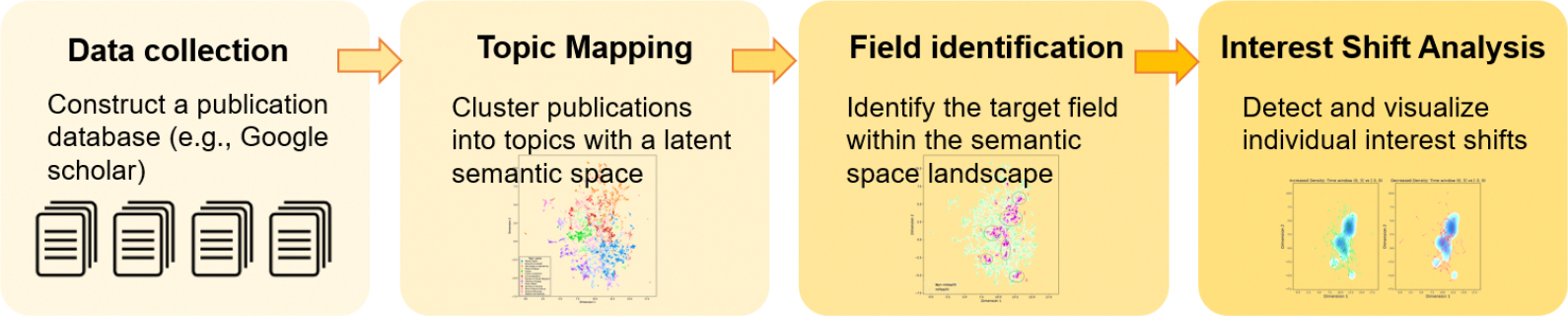
The proposed analytic framework for interdisciplinary training evaluation.

The emergence of LLMs and other advanced computational techniques has facilitated large-scale text analysis, offering new ways to uncover nuanced semantic structures within academic literature. These methods provide novel avenues for evaluating the broader intellectual and collaborative shifts resulting from training programs like mHTI. Motivated by these opportunities, our current study addresses these gaps by combining direct academic outcome data from mHTI participants (i.e., publications) with state-of-the-art techniques to provide a comprehensive analysis of the thematic landscape of mHealth research and the impact of interdisciplinary training programs. The proposed analytic framework is shown in Figure 1. By leveraging methods such as BERTopic modeling and LLM-based topic classification, this study aims to showcase the diversity of research topics conducted by interdisciplinary mHTI scholars, assess the relative significance of mHealth-related publications, and evaluate the influence of the mHTI program on scholars’ research trajectories. Furthermore, the proposed framework for tracking scholars’ research interest shifts is fully adaptable and can be transferred to the evaluation of other interdisciplinary training programs. This generalizability enables broader applications of our approach in assessing the long-term intellectual impact of training initiatives across domains. Furthermore, the proposed measurement framework for tracking scholars’ research interest shifts is fully adaptable and can be transferred to the evaluation of other interdisciplinary training programs.

## 3. Analysis Methods

### 3.1. Data Processing

The present study relies on publication records of scholars who participated in the Mobile Health Training Institute (mHTI). Given the relatively long duration from research ideation to publication [28], we focus on the 2015 to 2022 scholar cohorts to effectively capture the lagged impact of program training (excluding 2020 due to the COVID-19 pandemic). As a result, we collected a total of 229 scholars’ publicly available publications up to January 2024 from Google Scholar.

Google Scholar’s wide-ranging coverage across various disciplines, quick indexing, and easy accessibility [29] make it an essential tool for compiling thorough publication records for our analysis. However, collecting public publications through Google Scholar presents several challenges, due to name disambiguation and the need for complete publication records. To overcome these issues, we started by identifying scholars with clearly distinguishable personal profiles on Google Scholar. To improve search accuracy and increase the number of scholars with identifiable profiles, we supplemented verification searches with additional information such as institution and email domains. As a result, a total of 176 mHTI scholars were matched with their Google Scholar profiles, while the remaining 53 scholars, who could not be reliably identified due to insufficient or unclear information with the described approach, were excluded from the analyses. Table 1 shows the demographic information of the 176 mHTI scholars included in our analysis sample. The scholars primarily come from psychology, computer science, statistics, engineering, and data science, comprising 66% of the total. Additionally, most scholars are in mid-career, primarily assistant or associate professors.

Using the scholarly package [30] with Python 3.10.14, we systematically gathered publication data for each scholar, including article titles, abstracts, author lists, and publication years. Data collection resulted in a set of 19,083 publication records from Google Scholar. To ensure data quality, we removed records with missing or excessively short abstracts, keeping a final set of 16,580 publication records. This dataset served as the foundation for the topic modeling analysis elaborated in the subsequent subsection.

### 3.2. Topic Modeling of mHTI Scholars Based on LLM’s Semantic Latent Space

**Figure 2.**
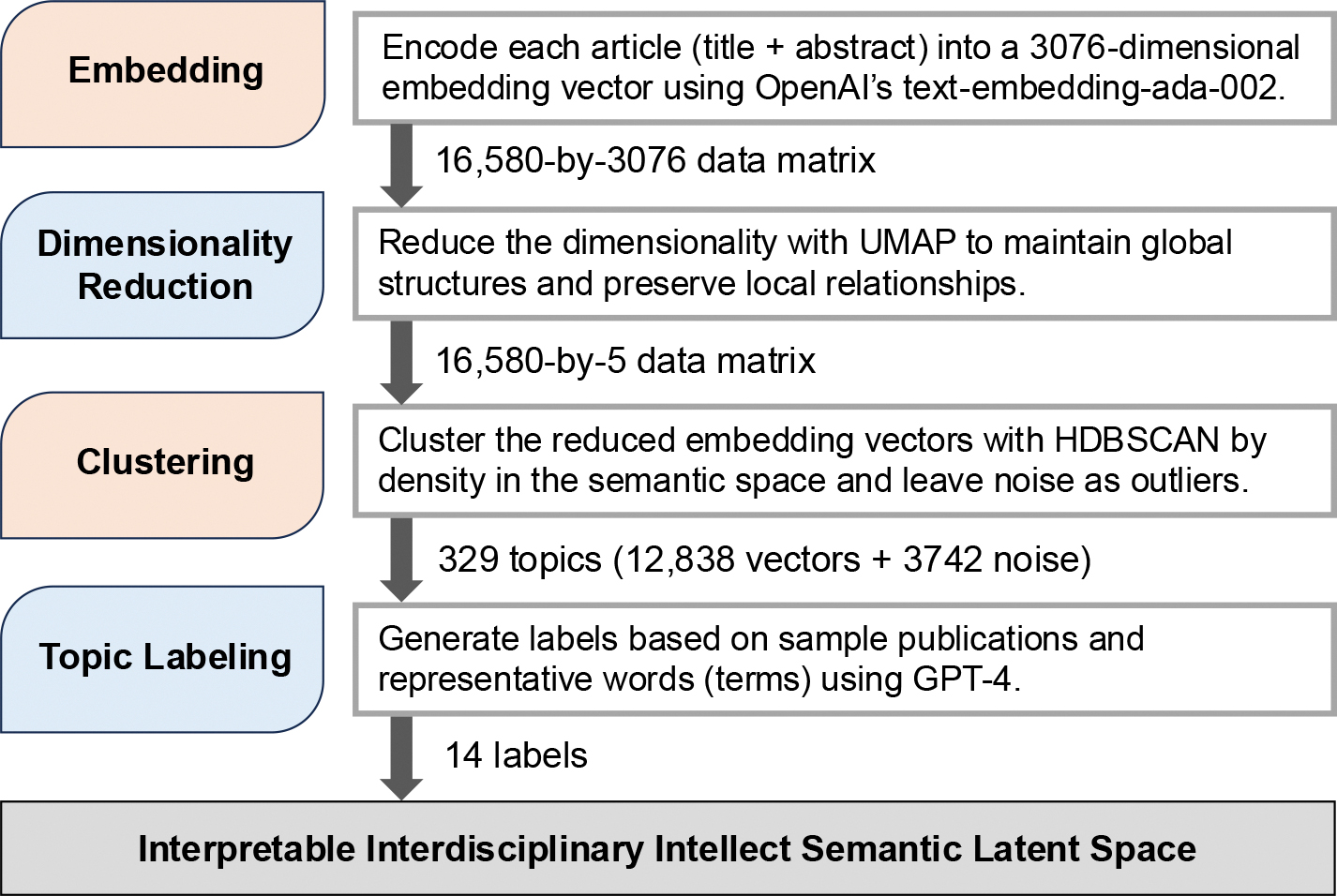
Topic modeling pipeline.

To address the first research question, we aim to identify scholars’ research fields of interest through topic modeling of their published works, a method widely used in scientific research analysis [19,31,32]. Specifically, our strategy is to integrate the BERTopic framework [20] with advanced large language models (LLMs, [33]) to establish a hybrid analytical pipeline as shown in Figure 2. The implementation of the topic modeling proceeds through three optimized steps, where LLMs are employed as neural topic summarizers [34] to generate human-interpretable labels that preserve conceptual granularity while maintaining semantic coherence with the original embeddings. We describe each of the steps in this section below.

#### 3.2.1. Semantic Embedding of Publication Titles and Abstracts Using LLM-Derived Representations

To transform the semantic meaning of text inputs into numerical inputs, the embedding vectors are constructed. An embedding is a numerical representation of text (or other data) in a high-dimensional vector space, which is then referred to as an embedding space. It ensures that similar texts have similar vector representations. According to the linear representation hypothesis, certain directions in the embedding space of a large language model correspond to specific, human-interpretable concept changes [35]. This means that by moving along these directions within the model’s representation space, we can effect predictable and meaningful alterations in the concepts represented by the model. Such embeddings are particularly useful for downstream tasks like clustering, classification, and search. Compared with traditional natural language processing models, LLMs offer a deeper contextual understanding, often surpassing earlier models and even approaching human-level performance across various tasks. Consequently, integrating LLMs into science of science research is highly valuable, where they not only provide novel methods for knowledge discovery, such as summarizing scientific literature, but also enhance existing text analysis workflows by offering a more robust embedding space, thereby improving the accuracy of tasks like topic modeling. We employ LLMs for multiple use cases in this study.

We adopt the general topic modeling pipeline proposed by BERTopic [20], augmented with LLMs to enhance both accuracy and interpretability. Specifically, we treat the combination of a publication’s title and abstract as a representative summary of its content. For each publication, we concatenate the title and abstract and encode them into a 3076-dimensional embedding vector for each publication using OpenAI’s publicly available advanced embedding model, text-embedding-ada-002. With 16,580 such embedding vectors, we proceed with the standard topic modeling process, which involves dimensionality reduction followed by unsupervised clustering. The resulting clusters are interpreted as distinct research topics.

#### 3.2.2. Dimensionality Reduction to Construct a Latent Semantic Space

Although the embedding vectors successfully preserve semantic information in numerical form, their high-dimensional nature complicates further analysis. To address the challenges posed by high dimensionality, we employ Uniform Manifold Approximation and Projection (UMAP, [36]) for dimensionality reduction. UMAP can not only preserve local relationships between data points but also partially maintain global structures in the reduced space. UMAP achieves this by determining local neighbors and constructing a proximity global graph among data points. This ensures that the clustering algorithm can operate effectively in a lower-dimensional space, avoiding the “curse of dimensionality” that often plagues high-dimensional data [37]. By reducing the 3076-dimensional embeddings to a more manageable number of dimensions, we strike a balance between computational efficiency and the preservation of meaningful semantic relationships. Additional parameter tuning details are provided in Appendix A.

#### 3.2.3. Clustering Publications in the Semantic Space

For clustering, we use the Hierarchical Density-Based Spatial Clustering of Applications with Noise (HDBSCAN, [38]), a method recommended within the BERTopic framework. HDBSCAN is particularly well suited for topic modeling due to its ability to identify clusters based on dense regions in the vector space, which is crucial for textual embeddings that often exhibit uneven or sparse distributions. Such sparse distributions may arise in data derived from interdisciplinary publications, where underlying uneven structures reflect disciplinary differences in the embedding vectors, with some papers concentrated on specific regions. Additionally, HDBSCAN effectively handles noise by labeling points that do not fit into any cluster as outliers, thereby improving the purity and interpretability of the resulting topics. This approach avoids the forced inclusion of unrelated documents into topics, ensuring that each topic remains coherent and meaningful.

The performance of the clustering is evaluated using the Silhouette score [39], which measures how similar an object is to its own cluster compared with other clusters. The Silhouette score for a single point is defined as

$$s(i)=\frac{b(i)\;-\;a(i)}{max\{a(i),b(i)\}},$$

where a(i) is the average distance from point i to other points in the same cluster, and b(i) is the smallest average distance from point i to points in a different cluster. The overall Silhouette score is the average of s(i) across all points, with higher values indicating better-defined clusters. Besides, HDBSCAN inherently produces unclassified publications (noise points) as part of its clustering approach. To balance cluster quality and classification coverage, we selected 11 as the minimum cluster size based on Silhouette score optimization while maintaining a reasonable proportion of unlabeled publications. (See Appendix B for parameter tuning details).

#### 3.2.4. Fostering Interpretation of Clusters

To interpret the identified clusters, we further employ the combination of HDBSCAN and GPT-4o (the state-of-the-art LLM available at the time of analysis) to generate descriptive labels and summaries for each cluster, that is, the identified topic.

**Figure 3.**
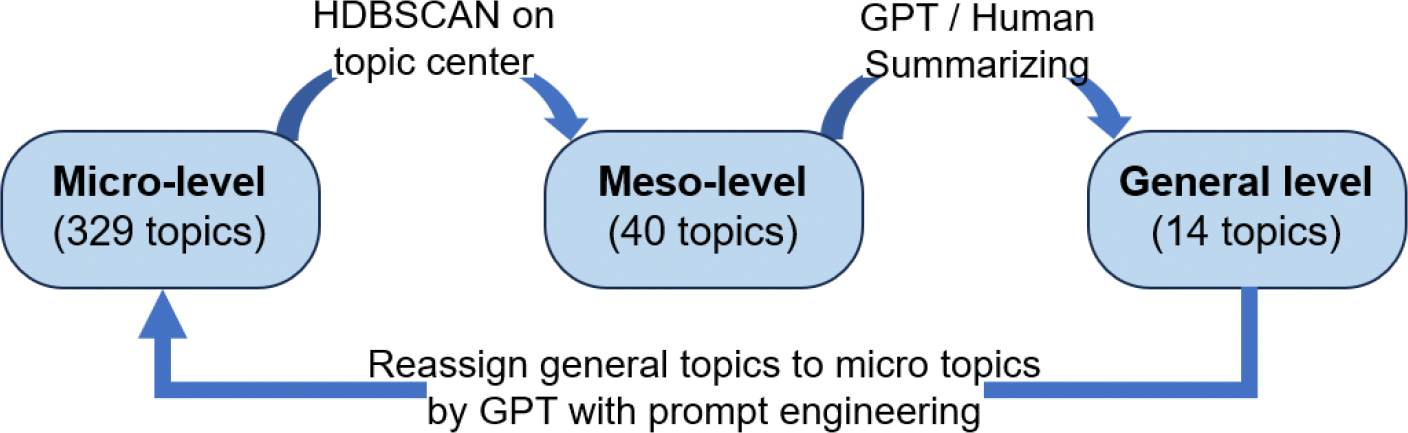
Flowchart of the hierarchical topic clustering process.

First, we apply HDBSCAN to re-cluster the previously obtained topics and gain the resulting meso-level clusters. This is achieved by calculating the centroid of each topic’s embedding vectors, averaged across all member publications (see Appendix D for a detailed description of the partial mapping from meso- to micro-topics). Second, to obtain general labels/topics that are interpretable, the meso-level clusters are further refined with GPT-assisted summarization and human validation. Lastly, with the general labels/topics, we use GPT-4o to assign each of the micro-topics to a single corresponding general label/topic. In the prompt engineering process, we provide GPT-4o with the label and description of each micro-topic, along with 10 randomly sampled article titles/abstracts from that topic, and representative keywords extracted via BERTopic’s TF-IDF measure. See Appendix C for the details of the prompt. This approach ensures that topics are both statistically robust and semantically grounded in mHealth research. A flowchart of this process is provided in Figure 3.

### 3.3. Labeling mHealth-Related Topics

To answer the second research question, we further focus on the distribution of mobile health (mHealth) articles within the semantic space. The key challenge lies in accurately identifying mHealth-related articles, given the highly interdisciplinary nature of the field. To address this, we employ a GPT-based approach to label mHealth articles. Specifically, we use the previously obtained 329 topics, along with their descriptions and 10 randomly selected article titles and abstracts from each topic, as input to GPT. The model is tasked with determining whether a topic is mHealth-related or not. To ensure precision, we carefully design the prompt to classify only topics and their internal articles that are strongly related to mHealth. This approach yields highly reliable mHealth-related topics. Due to the granularity of the topics obtained from topic modeling, we find that this topic-level identification method provides highly accurate predictions. Consequently, we use this method to label mHealth articles and provide a detailed interpretation of their distribution in the semantic space.

### 3.4. Measuring Migration of Scholars’ Research Interests

Building on the meaningful semantic space and mHealth article labels obtained from the previous steps, we aim to examine how scholars’ research interests evolve after they completed in the mHTI program. Instead of simply measuring changes in the number of mHealth publications, we leverage the rich information in the semantic space to detect shifts in scholars’ research topics. Specifically, we examine whether or not the scholars move toward the regions of the semantic space where mHealth articles are concentrated.

To achieve this, we first model the probability distribution of mHealth articles in the semantic space using kernel methods. For computational convenience, we perform this estimation directly in the two-dimensional semantic space used for visualization. We use a Gaussian kernel with a bandwidth of 0.8. The kernel density estimate (KDE) at a point x in the semantic space is given by

$$f(x)=\frac{1}{nh^{d}}\sum_{i=1}^{n}K\left(\frac{x\;-\;x_{i}}{h}\right),$$

where n is the number of mHealth articles, h=0.8 is the bandwidth, d=2 is the dimensionality of the semantic space, and K(·) is the Gaussian kernel function defined as

$$K(u)=\frac{1}{\sqrt{2\pi }}exp\left(-\frac{1}{2}\|u{\|}^{2}\right).$$

Next, we represent each scholar’s research interests as an average vector of the embeddings of their published articles. For a scholar with m articles, their average research interest vector $\bar{v}$ is computed as

$$\bar{v}=\frac{1}{m}\sum_{j=1}^{m}v_{j},$$

where $v_{j}$ is the embedding vector of the j-th article. We define the year of participation in the mHTI training as year 0 and calculate the average research interest vector for each scholar over three-year intervals. Specifically, we compute the average interest vector for the three years following training (interval (0, 3]).

Finally, we determine whether the scholars’ average research interests shift toward the regions of the semantic space with higher mHealth probability density. For each scholar, we compute the difference vector Δv between their post-training average vector ${\bar{v}}_{\text{post}}$ and their pretraining average vector ${\bar{v}}_{\text{pre}}$:

$$\Delta v={\bar{v}}_{\text{post}}-{\bar{v}}_{\text{pre}}.$$

We then evaluate whether this difference vector results in a movement toward the regions of higher mHealth probability density. Specifically, we calculate the change in probability density between the pretraining average vector ${\bar{v}}_{\text{pre}}$ and the post-training average vector ${\bar{v}}_{\text{post}}$. The change in probability density Δf is given by:

$$\Delta f=f\left({\bar{v}}_{\text{post}}\right)-f\left({\bar{v}}_{\text{pre}}\right),$$

where f(x) is the mHealth probability density function estimated using the kernel method. A positive value of Δf indicates that the scholar’s research interests have shifted toward the regions of the semantic space with higher mHealth density. This approach allows us to assess the impact of mHTI on scholars’ research trajectories.

## 4. Results

### 4.1. Research Topics: Semantic Space for mHTI Scholar Publications

**Figure 4.**
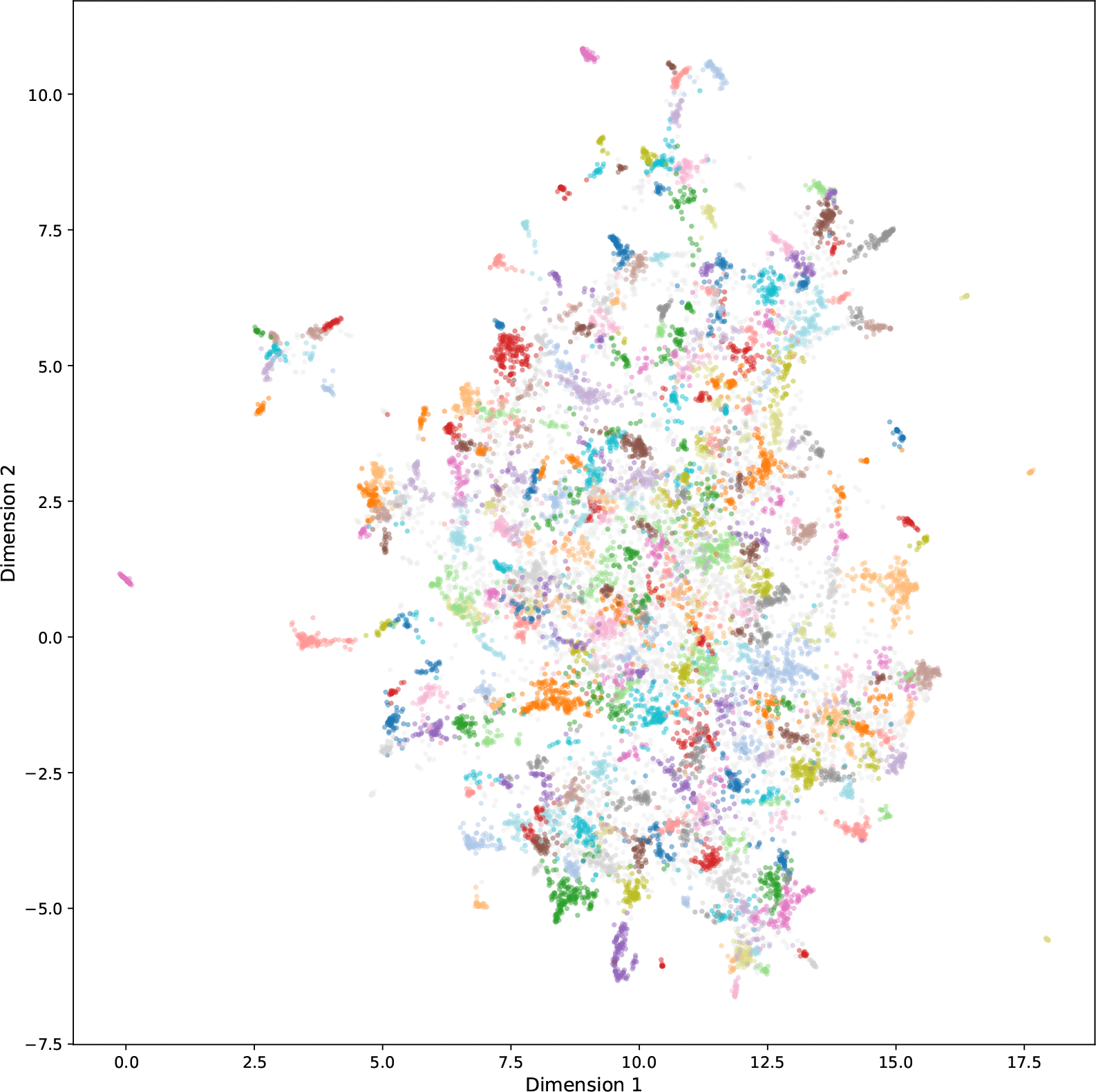
Semantic space for the mHTI scholars’ publications. Different colors indicate 329 topic labels. Unlabeled topics are coded in light gray.

Following the pipeline described in Section 3.2, a total of 12,838 of the 16,580 articles are clustered into 329 topics, leaving 3742 articles as noise in the semantic latent space. Figure 4 presents a 2D UMAP visualization of the high-dimensional embedding vectors, color-coded according to the 329 topic labels generated by our topic modeling process. Each dot represents a publication of the mHTI scholars. The color is coded by research topic, while unlabeled topics (classified as noise by the HDBSCAN algorithm) are coded in light gray.

**Figure 5.**
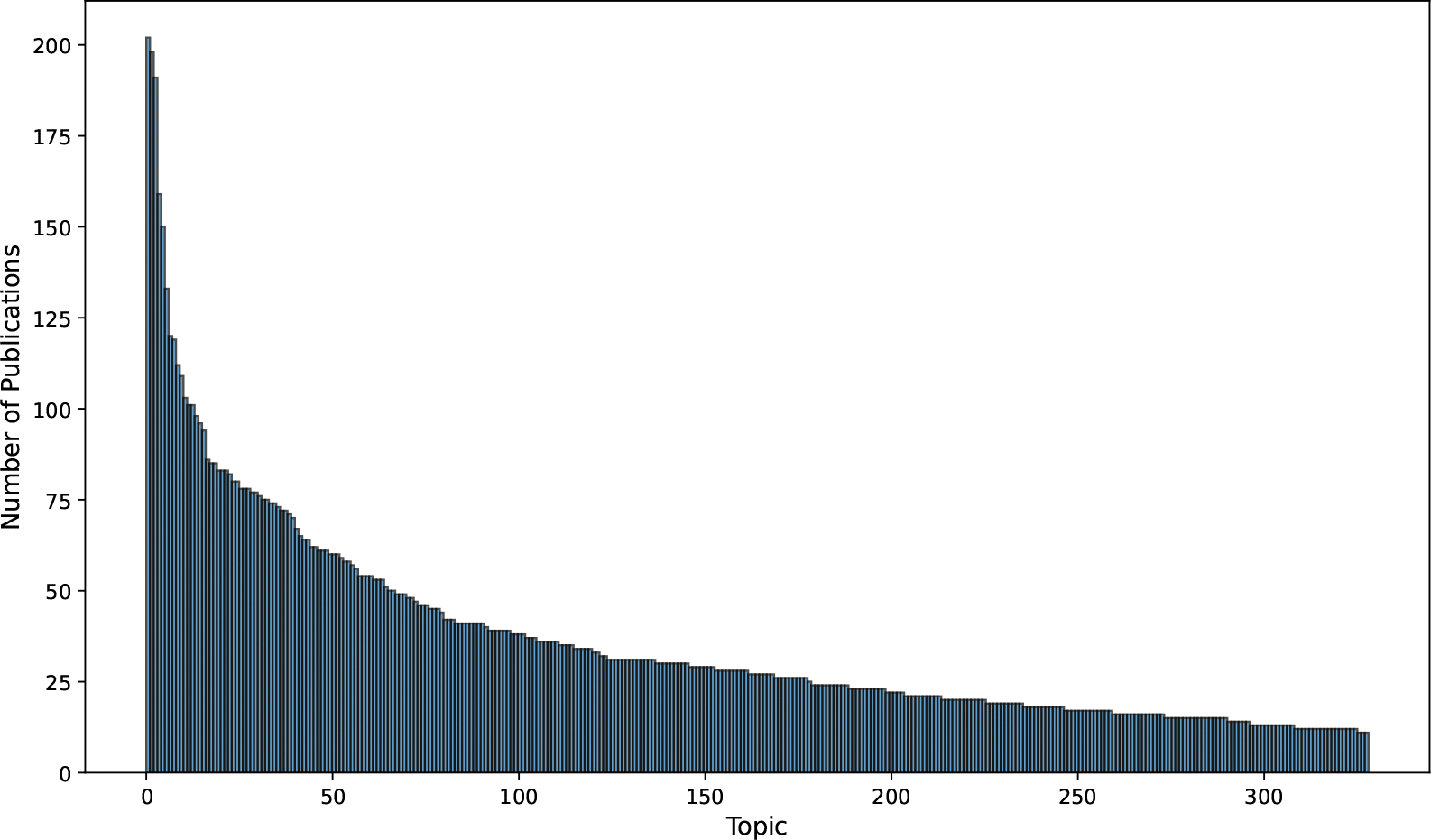
Distribution of the sizes (number of publications) of the 329 topics.

The distribution of the topic size is presented in Figure 5. Only 4.0% of the topics include more than 100 articles, while 45.6% of the topics include fewer than 25 articles. The distribution of the topic size indicates that mHealth scholars works on diverse research topics, while many such topics are studied by a relatively small number of scholars, meaning they are new or at its developing phase. For example, one cluster with 11 articles primarily focuses on the use of AI in healthcare, with all published after 2020.

To further illustrate, we list the top five topics with the largest cluster sizes in Table 2 and five topics with smallest cluster sizes in Table 3, along with their GPT-generated descriptions. Empirical manual inspection confirms that the assigned documents align well with their respective topic labels. We provide a complete table of 329 topics in the Supplementary Materials.

#### Remark 1.

*We observed that during the process of topic and description generation, GPT sometimes produced topic descriptions that include specific geographic names, such as disease research in a particular country, which is not shared by all papers in the cluster and is prone to change when another sample is selected. Therefore, we designed an additional prompt to refine the generated labels, removing specific geographic information from the topic descriptions. The prompt is also provided in the* Appendix C.

**Figure 6.**
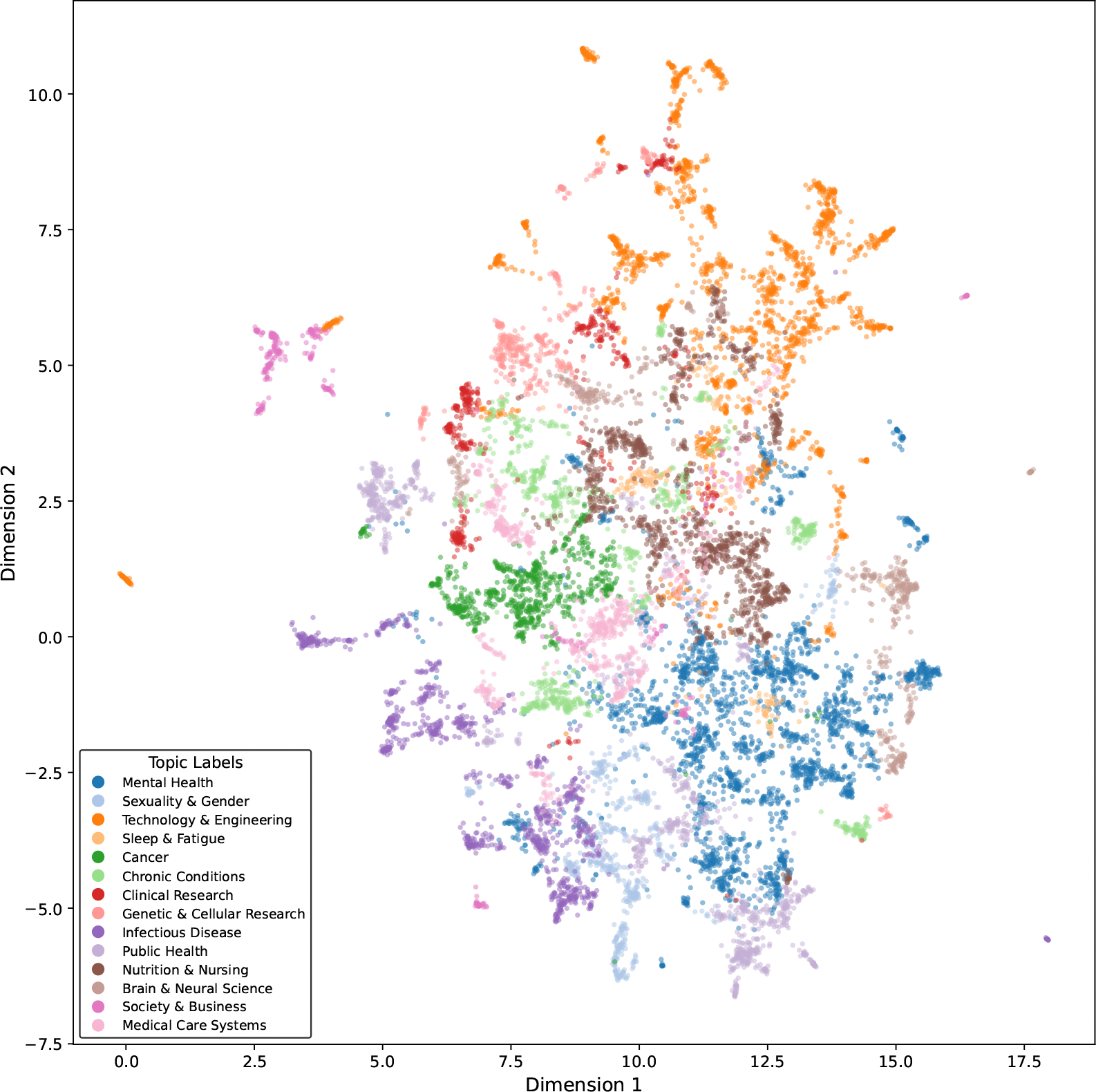
Semantic space for the mHTI scholars’ publications, color-coded by 14 general labels.

Due to the complexity of interpreting 329 micro-topics, we further cluster the topics at a higher level to facilitate the interpretation of the semantic space. With the combination of HDBSCAN and GPT-4o as well as manual human validation, 14 general topics are identified. We use GPT’s contextual understanding to directly assign general topics to the 329 micro-topics, rather than relying on clustering algorithms. For this approach, it is crucial that the GPT-assigned general topic labels align with the semantic space. Specifically, microtopics belonging to the same general topic should be located in close proximity within the semantic space. The final result, visualized in Figure 6, demonstrates strong alignment between the location of semantic spaces and the assigned general topics, validating the coherence of our topic modeling approach. These 14 general topics directly address Q1, highlighting the primary research topics of the mHTI scholars.

The visualization offers rich insights into the semantic space for the mHTI scholar’s publications. Notably, the effectiveness of the general clusters is validated by the spatial distribution of topics. For instance, technology and engineering topics (dark orange) are concentrated in the upper-right quadrant, while mental health and public health topics (dark blue) are located in the lower region. Clinical diseases such as cancer (dark green) and chronic conditions (light green) are clustered in the middle-left area, and social sciences (dark pink), positioned in the upper-left quadrant, are relatively distant from other disciplines. Topics like nutrition, nursing (brown), and healthcare systems (light pink) occupy the central region of the semantic space, suggesting their broad relevance across multiple fields.

The spatial arrangement of the general topics also highlights interdisciplinary intersections. For example, infectious disease research (dark purple) lies at the intersection of clinical medicine (red) and public health (light purple), reflecting its dual relevance to both fields. Similarly, topics related to sexuality and gender (light blue) are situated between public health (light purple) and mental health (dark blue), aligning with their prominence in discussions within these domains. Meanwhile, technology topics (dark orange) are closely intertwined with clinical medicine (red), healthcare systems (light pink), nursing (brown), and neuroscience (light brown), underscoring their pervasive role in advancing medical research.

This spatial coherence not only validates the robustness of our topic modeling approach but also provides an intuitive way to explore the relationships between research areas.

### 4.2. Mobile Health-Related Publications from mHealth Scholars in the Semantic Space

We further examine the distribution of mHealth research within the semantic space. With the method elaborated in the Methods Section, we identify mHealth topics and corresponding mHealth publications with the assistance of GPT-4o.

**Figure 7.**
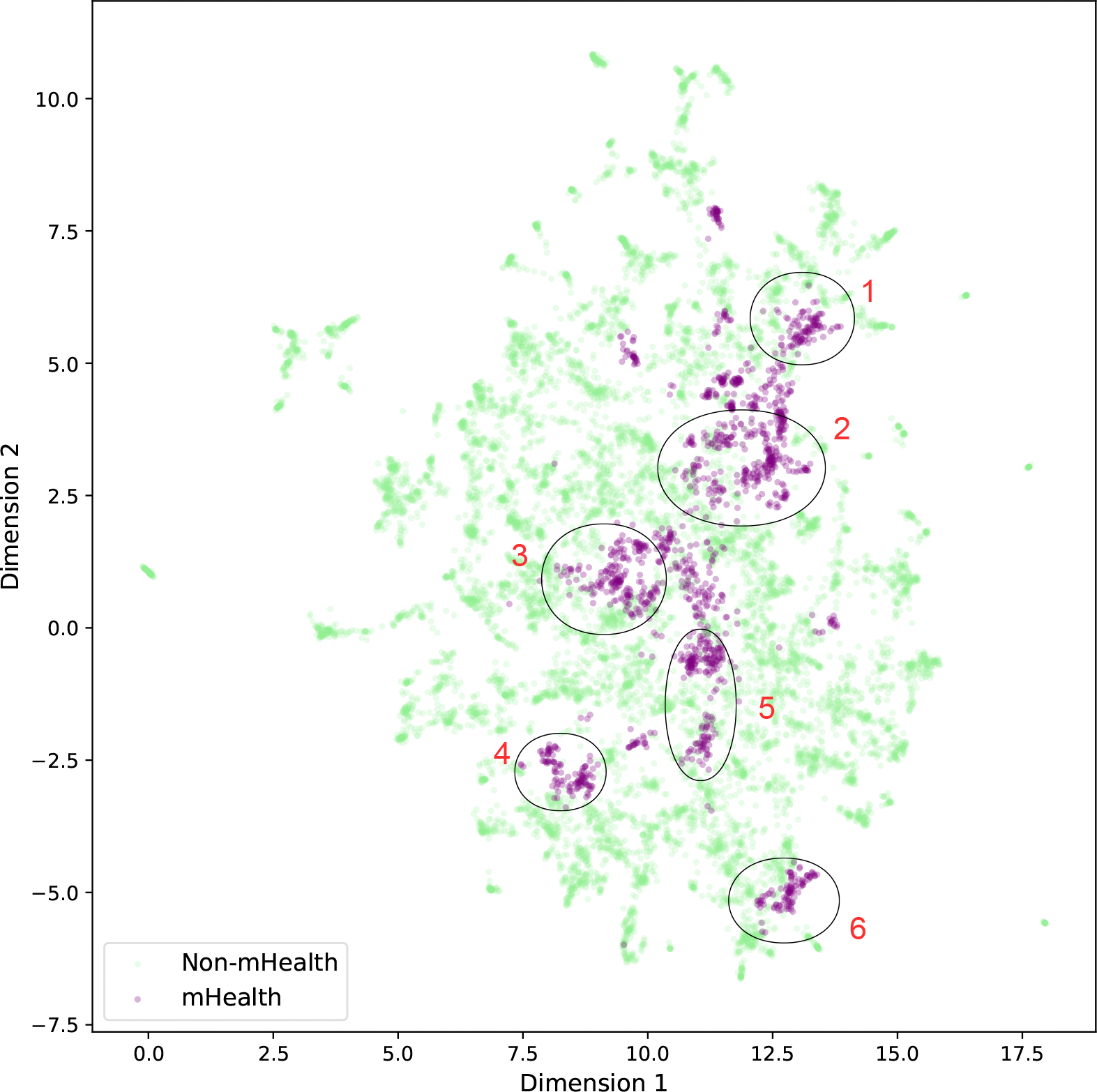
Distribution of mHealth papers in the semantic space for the mHTI scholars’ publications. Purple indicates mHealth topics, and green indicates non-mHealth topics. Numbers 1 to 6 indicate six regions of mHealth topics for illustration.

Figure 7 plots publications with mHealth tags in a 2D UMAP space, revealing an intriguing pattern: mHealth-related articles are predominantly located in the central region of the semantic space, connecting various general topics. We further zoom in on six humanly marked regions in the 2D semantic space and examine their corresponding micro-topics, listed in Table 4.

We note that regions 1 and 2 are close to the general topic of technology, with their micro-level topics primarily focusing on mHealth-related research in technological development, algorithms, and hardware, emphasizing technological innovation. That is, these two regions appear to represent the closest intersection between mHealth and technology. Region 3, located at the center of the semantic space, leans toward medical topics such as clinical, mental health, and nursing. Its subtopics show a focus on practical scenarios of mHealth, including, but not limited to, psychological care and intervention, as well as chronic disease management. Region 4 tends toward the public health and infectious disease general topics, aiming to leverage mHealth for the prevention, control, and detection of infectious diseases. Region 5 is more aligned with the mental health general topic in the lower-right corner of the semantic space, with its subtopics directly related to mental health. Finally, as a distinct area, region 6 focuses on smoking cessation. Although the GPT-generated description of this subtopic does not explicitly mention mHealth, a detailed examination of the articles within this topic reveals a strong emphasis on the practical value of mHealth in smoking cessation interventions, leading to its classification as an mHealth-related topic.

Through this analysis, we gain insights into the intersections between mHealth and various broader fields. The precise identification of mHealth subtopics by GPT, as well as the consistency between the distribution of these subtopics in the semantic space and their corresponding content, further validates the effectiveness of using semantic space clustering for topic modeling.

### 4.3. Research Interest Shift of Scholars

**Figure 8.**
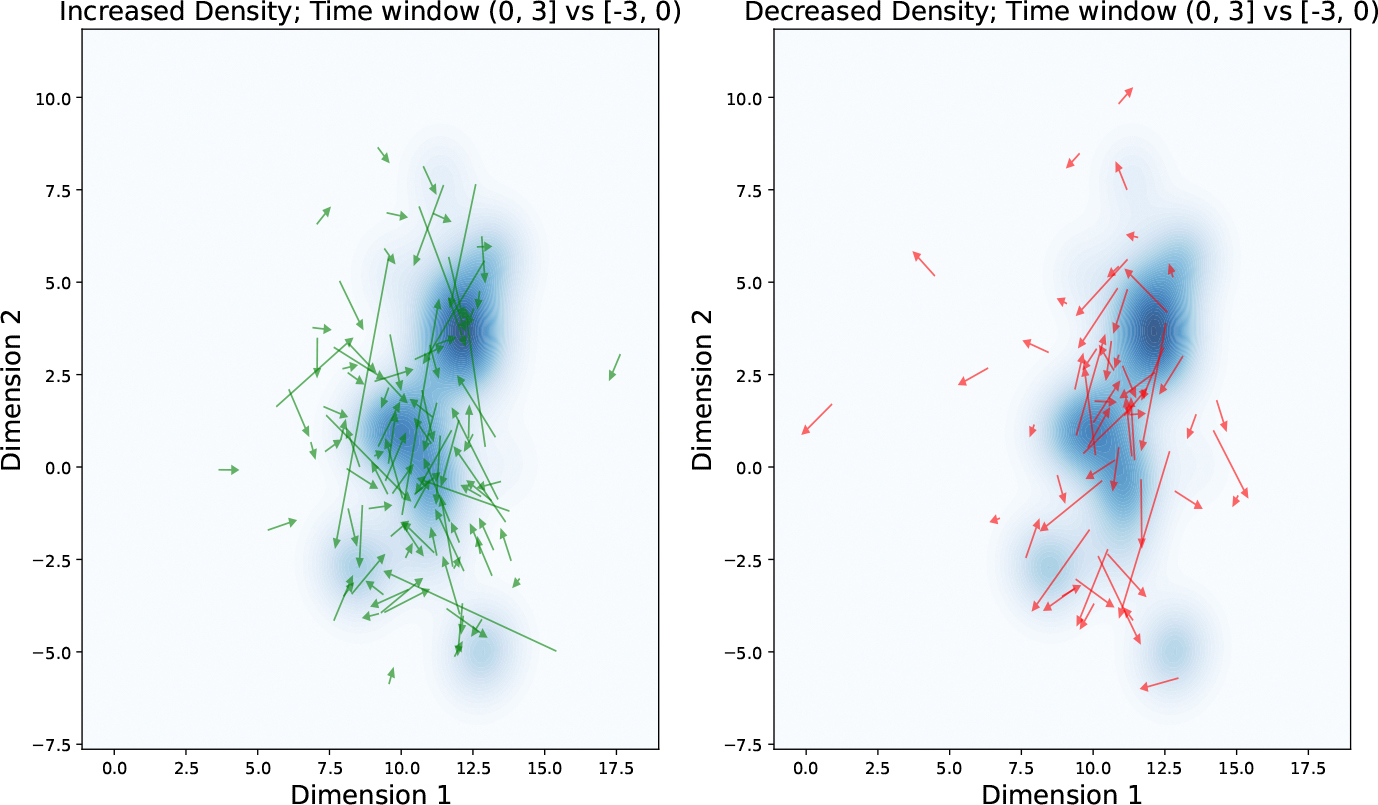
mHTI scholars’ migration in semantic space. The left subplot shows scholars moving toward higher probability density regions (dark blue), while the right subplot shows scholars moving away from the high-density regions. The arrows indicate migration direction.

Based on the semantic space of mHTI scholars and the previously identified distribution of mHealth-related articles, we examine how participation in the mHTI program influences changes in scholars’ research topics over time. Using kernel density estimation, we model the distribution of mHealth publications within a two-dimensional semantic space. For each scholar, we compute an average research interest vector within three-year time windows. Specifically, we define the post-mHTI period as (0, 3]—the three years following participation—and compare it with the pre-mHTI period, defined as [−3, 0). The relative positional changes are visualized in Figure 8. In this figure, the tail of each arrow marks the centroid of a scholar’s publication topics before mHTI, and the arrowhead indicates the centroid after mHTI. Each arrow thus represents the direction and magnitude of topic migration. As shown, some scholars shift toward regions of higher mHealth topic density (darker blue), while others move away from these regions during the same time window.

The probability density function across the subplots closely matches the distribution of mHealth-related topics, indicating that our kernel fitting effectively captures the distribution of mHealth in the semantic space. A total of 163 scholars who have publications in both three-year time windows were included for this analysis. Results reveal that about 63.8% of scholars move closely toward mHealth regions in the semantic space after participating in mHTI. Combined with the observation in Figure 8, we note that the number of scholars moving toward mHealth-related semantic regions consistently exceeds those moving away. This means that, although most scholars are outside of the medical field, as shown in Table 1 in the mHTI scholar pool, mHTI promotes the attractiveness of the mHealth field to scholars.

To establish statistical significance, two null models were developed based on random movement assumptions in the constructed semantic space. Both models initialize scholars at their average semantic locations derived from [−3,0) year publications. The empirical displacement vectors Δ**v** were calculated between these starting positions and positions derived from (0,3] year publications, maintaining consistency with the previous trajectory analysis.

The first null model preserves the observed displacement magnitudes ||Δ**v**|| while randomizing movement directions. The second model introduces additional variability by randomizing both direction and distance, sampling displacements uniformly from [0, 1.2||Δ**v**||]. For each simulation, movement outcomes were evaluated by comparing pre- and post-movement KDE values for mHealth topic distributions.

**Figure 9.**
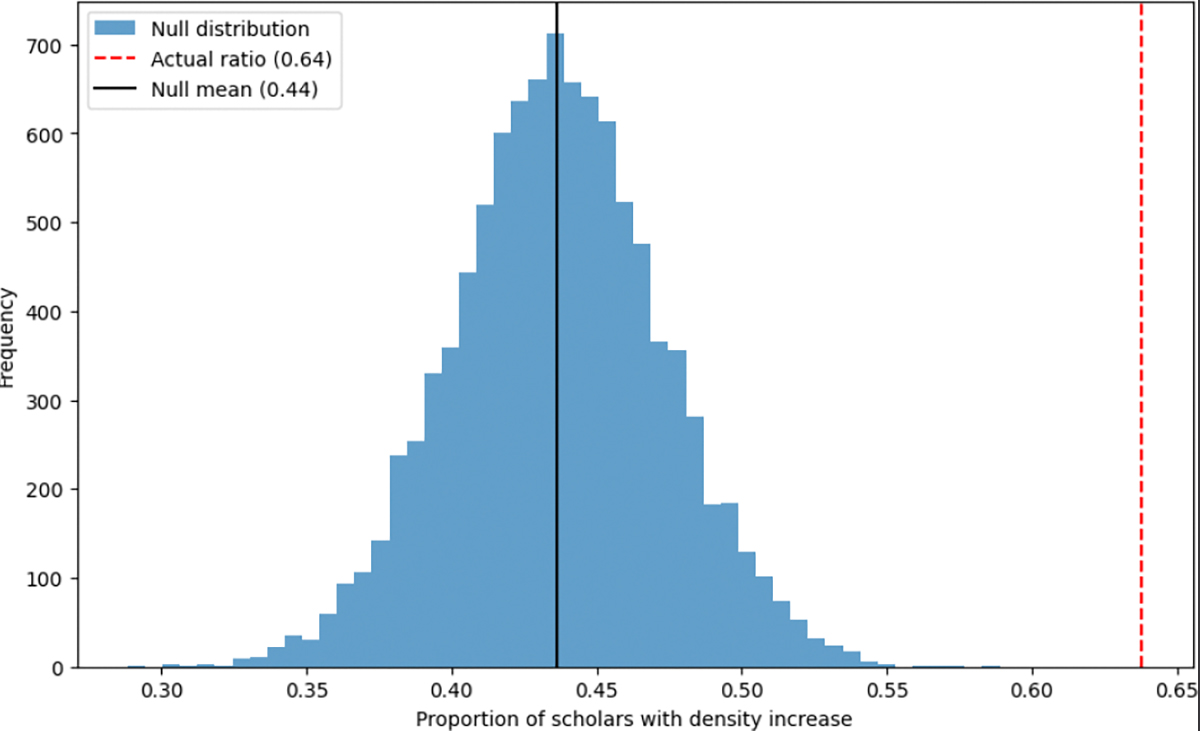
Comparison of the actual increase ratio with the null distribution under a fixed distance setting.

**Figure 10.**
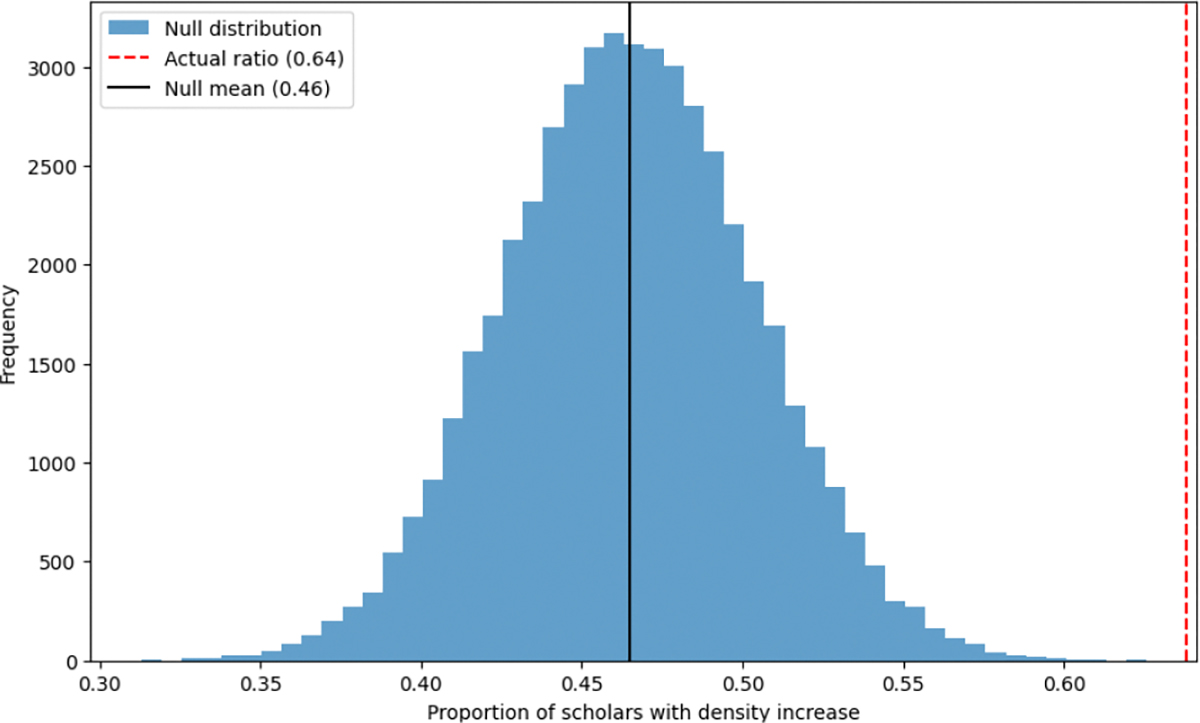
Comparison of the actual increase ratio with the null distribution under a random distance setting.

The fixed-distance model (10,000 iterations) and variable-distance model (50,000 iterations) both yielded null distributions where the observed 63.8% attraction rate fell in the extreme upper tail (*p* < 0.001, see Figures 9 and 10). This confirmation across both randomization approaches demonstrates that the observed trajectory shifts toward mHealth topics significantly exceed chance expectations, substantiating the program’s influence on research directions.

**Figure 11.**
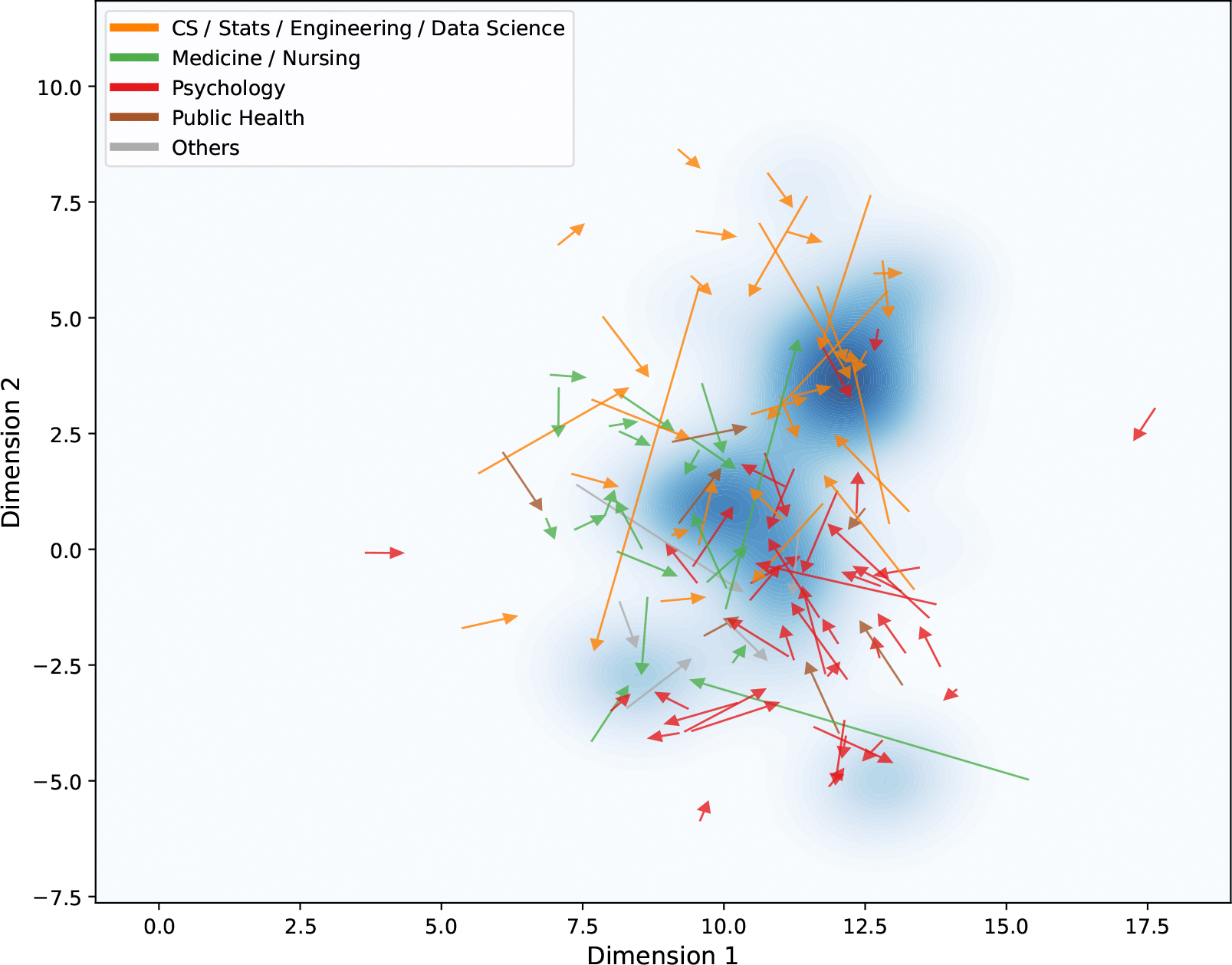
Disciplinary-based migration patterns of mHTI scholars toward mHealth topics in semantic space, with time window [−3, 0) vs (0, 3]. The arrows indicate migration direction.

For scholars whose research interests shifted toward mHealth, we further examine demographic-level topic migration by analyzing patterns based on scholars’ disciplinary backgrounds (see Figure 11). Overall, most scholars’ research trajectories remain anchored in their original disciplines. For example, scholars from technology-related fields tend to cluster in the upper-right quadrant of the semantic space, while those from medical or nursing backgrounds are concentrated near the center. Scholars with psychology backgrounds are typically located in the lower-right quadrant, which is densely populated with mental health publications.

As shown in Figure 11, during the given relatively short time windows (three years before and after participating in mHTI), scholars often migrate toward nearby high-density regions of mHealth-related topics—represented by the darkest blue areas. These “peaks” indicate regions in the semantic space where mHealth publications are most densely concentrated, reflecting prominent or well-established subfields. Rather than shifting broadly across disciplines, scholars tend to move toward mHealth topics that are semantically close to their existing research interests. This pattern suggests a tendency toward disciplinary continuity, where scholars integrate mHealth themes that align with their prior expertise. We also observe instances of long-distance migration in the figure, indicating more substantial shifts in research direction following participation in mHTI. These cases reflect a deeper reorientation of scholarly focus and highlight the program’s potential to catalyze significant topic-level transformations.

### 4.4. Cohort and Disciplinary Analyses of mHealth Publication Proportion Shifts

To further substantiate the observed shift in research focus toward mobile health among mHTI participants, we conduct traditional comparative analyses of scholars’ pre-versus post-training mHealth publication ratios across cohort years and disciplinary subgroups. We maintain the three-year windows before training ([−3, 0)) and after training ((0, 3]) as our observation periods. Using cohort years and research disciplines as subgroup indicators, we present changes in the proportion of mHealth publications among all publications for scholars in each subgroup. Positive changes in this proportion may suggest increased research interest in mHealth among participants.

Specifically, our analysis is still based on 12,838 articles with well-identified topics. For each scholar, we calculated the percentage of mHealth publications relative to their total publications during both the pre- and post-training three-year periods. For each subgroup, we then report the mean values of these percentages, together with the validated number of scholars (scholars who have identified publications in both [−3, 0) and (0, 3] time windows).

Tables 5 and 6 reveal a widespread increase in mHealth publication proportions following training across multiple cohorts and disciplines. Cohort-level analysis demonstrates consistent positive growth in mean proportions for cohorts with complete data (2015–2019). For the 2021 and 2022 cohorts, we observed more modest changes. This attenuated effect likely reflects incomplete data collection, as our dataset extends only through 2023—insufficient to capture the full 3-year post-training window for these recent cohorts. Disciplinary comparisons indicate substantial increases in engineering, medical, and psychological sciences, with public health showing more modest gains and other disciplines displaying merely negligible effects. However, the number of scholars in other disciplines is limited, which could lead to random fluctuations for this subgroup.

## 5. Conclusions

### 5.1. Summary and Discussion

This study presents a hybrid analytical framework that pipelined LLM-derived semantic embeddings, UMAP dimensionality reduction, and HDBSCAN clustering to map the interdisciplinary landscape of mobile health (mHealth) research and the impact of the mHTI on the trained scholars’ research trajectories. We identified 329 micro-topics, grouped into 14 high-level themes, which delineate how mHealth receives influence from diverse disciplines, such as technology, clinical medicine, public health, mental health, and social sciences. Our method employed LLM-based topic classification to isolate mHealth-specific topics within the semantic latent space, showing that mHealth research serves as a central nexus across diverse disciplines. This result underscores the importance of creating “boundary space” where scholars can reconfigure existing expertise into novel research vectors. Furthermore, kernel density estimation revealed that around 64% of mHTI participants shifted their research focus toward mHealth areas within three years of the program participation, demonstrating that structured training initiatives can effectively stimulate cross-disciplinary research engagement. Our study affirms the value of the targeted training program, mHTI, in guiding scholarly inquiry in emerging fields like mHealth, while providing a scalable and nuanced framework for analyzing the evolution of interdisciplinary research domains.

Compared with previous approaches that rely on qualitative data, self-reported surveys, or coarse quantitative indicators such as publication or grant counts, our study offers a more objective and scalable framework based on publication content to track long-term shifts in research interests. Unlike earlier work on the mHTI program that emphasizes short-term communication dynamics through network modeling [9], our study focuses on content-level changes using semantic analysis. Together, these contributions demonstrate the potential of computational text analysis to uncover long-term intellectual shifts and provide new insights into how interdisciplinary training programs shape the direction and integration of scholarly work.

### 5.2. Limitations and Future Directions

While this study advanced understanding of mHealth research dynamics of the mHTI scholars, several limitations should be noted: First, topic granularity was determined internally via Silhouette scores, an unsupervised approach that balances cluster coherence but risks over-specificity or over-generalization depending on sample characteristics. As a validation of our proposed method, we compared our results with those generated by NOMIC Atlas (https://atlas.nomic.ai/), a state-of-the-art topic modeling and visualization tool. Although NOMIC’s clustering and labeling processes are not open-source, it produced 256 topics with a minimum cluster size of 11 when applied to our embedding vectors. This outcome is comparable to our results in terms of topic count and cluster size, validating the robustness of our approach. While minor performance differences may stem from NOMIC’s noise handling and topic merging methods, the overall consistency between the two methods reinforces the reliability of our findings. In addition, further validation of the alignment between LLM-generated topic labels and the associated publications would strengthen the credibility of the results. In the current study, this validation was performed by a single human evaluator from the research team. Incorporating multiple independent raters in future work would enhance objectivity and enable formal assessment of inter-rater reliability.

Second, our analysis focused on the publications by a curated sample of mHTI-affiliated scholars; although practical for tracking training impacts, it may reflect institutional or selection biases. The resulting semantic space reflects topic configurations within this community rather than the broader mHealth field.

Third, our kernel-based trajectory method assumes monotonic proximity gradients in the semantic probability density landscape; however, interdisciplinary scholars often navigate multipeak regions, as shown in Figure 8. When a scholar’s research interest shifts from one mHealth-related peak to another, it becomes challenging to determine whether they are moving closer to or away from mHealth. However, we note that in our analysis, scholars identified as moving closer to mHealth (left subplot in Figure 8) are less likely to switch between mHealth-related intersections, as the green arrows are mostly distributed around the peaks, with fewer cases of arrows pointing from one peak to another. In contrast, for scholars identified as moving away from mHealth (right subplot in Figure 8), we observe several scholars migrating between peaks in the upper-right corner, and they might have been misclassified as moving away from mHealth. As a result, it is possible that we might have underestimated the number of scholars moving towards mHealth. If we included scholars switching between peaks, the proportion of scholars moving closer to mHealth would likely be higher.

Fourth, this study faces limitations related to data sources. We rely solely on Google Scholar to collect publication data for scholar matching and comprehensive publication coverage. This choice is driven by the interdisciplinary nature of mHealth research, which spans medicine, public health, psychology, computer science, engineering, and the social sciences. We focus on tracking shifts in scholars’ research interests rather than evaluating their research productivity. From this perspective, a wide range of academic outputs, including preprints (e.g., arXiv) and conference proceedings, are relevant indicators of evolving interests. Google Scholar offers broad coverage of these outputs and mitigates name ambiguity across data sources. That said, our framework is flexible and can incorporate additional bibliographic sources, ideally with manual verification, to further enhance accuracy. In addition, the availability of mHTI participant data begins in 2015, and our data collection ends in 2023. This time range limits our ability to observe longer-term research interest trajectories. As more longitudinal data become available in the future, our framework can be readily extended to support more comprehensive long-term analyses.

Despite these limitations, the present study contributes to the science of science by offering a robust framework for understanding the dynamics of knowledge production in interdisciplinary fields like mHealth. Building on these insights, several avenues remain for future studies. First, the kernel-based method used to track scholars’ research trajectories could be enhanced to better account for cross-disciplinary shifts. Second, future studies could expand the scope of analysis to include a broader range of interdisciplinary fields beyond mHealth. Investigating how training programs like mHTI influence participating scholars’ research trajectories in other domains could provide comparative insights into the mechanisms of interdisciplinary collaboration and knowledge production. Furthermore, longitudinal studies tracking scholars over extended periods could offer a deeper understanding of the long-term impacts of training programs on research interests and career trajectories.

Additionally, the current research focused more on a view of program evaluation, investigating how mHTI impacted the research trajectories of scholars. An important future research question could be how the mHealth field evolves with the infusion of fresh perspectives from scholars across different disciplines. Specifically, how do insights and knowledge from various fields shape mHealth, and what unique research topics emerge as a result?

Finally, the proposed workflow, which leverages LLM and semantic trajectory analysis, offers a scalable and interpretable approach to measuring long-term shifts in research interests. While this study focuses on the development and demonstration of a computational pipeline, future work could enhance its analytical depth through the integration of additional methods. Incorporating elements of causal inference, such as introducing a control group, would allow for a more rigorous evaluation of the program’s direct impact on research trajectories. At the same time, qualitative methods, including interviews or surveys with program participants, could enrich the analysis by capturing scholars’ personal experiences and perspectives on how the training influenced their intellectual focus and collaboration patterns. Together, these extensions would support a more holistic understanding of interdisciplinary research dynamics and the role of training programs in shaping them.

By addressing these directions, future research can build on the findings of the current study to further advance our understanding of interdisciplinary research dynamics and the mechanisms underlying knowledge production in fields like mHealth.

## Supplementary Material

Supplementary Material

**Supplementary Materials:** The following supporting information can be downloaded at https://www.mdpi.com/article/10.3390/app15116252/s1.

## Figures and Tables

**Table 1.** Demographic distribution of the 176 mHTI scholars (2015–2022) included in our analysis.

Variable	Frequency	Variable	Frequency

**Cohort**		**Career stage**	
2015	23	Early-career	55
2016	24	Mid-career	110
2017	30	Late-career	8
2018	19	Others	3
2019	25	**Gender**	
2021	29	Female	107
2022	26	Male	69
**Discipline**		**Region**	
CS/Stats/Engineering/Data science	53	Midwest	37
Medicine/Nursing	35	Northeast	49
Psychology	64	Southeast	30
Public health	15	Southwest	12
Others	9	West	32
		Others	16

**Table 2.** Top 5 largest topics, along with their descriptions and sizes.

Topic	Description	Size
Eating Disorders Research	Investigation of eating disorder symptoms, interventions, and impacts across various populations.	202
Pediatric Asthma Management	Focus on improving techniques, adherence, and care outcomes in children with asthma.	198
Virtual Reality Assessment	Evaluating cognitive functions using VR technology improves accuracy and ecological validity.	191
Alcohol Use in HIV	Exploring impacts and patterns of alcohol use among HIV patients.	159
Behavioral Weight Loss	Focuses on interventions and strategies to promote weight loss in diverse populations.	150

**Table 3.** Top 5 smallest topics, along with their descriptions and sizes.

Topic	Description	Size
Adolescent Disaster Mental Health	Examines PTSD, depression, and substance use in youths post-tornado exposure.	12
Self-Aware Systems	Systems with computational self-awareness for enhanced autonomy, adaptability, and performance in dynamic environments.	12
Human–Robot Interaction	Analyzing empathy, trust, and intention estimation in collaborative human–robot environments.	11
Principal Stratification	Investigating surrogate endpoints and causal estimands in vaccine trials using statistical methods.	11
AI Trust Healthcare	Building clinician trust in AI for effective integration in healthcare systems.	11

**Table 4.** Micro-mHealth-related topics of the six regions marked in the semantic space. For regions 1 to 5, three micro-topics were randomly selected from each region. In region 6, only a single micro-topic was identified as an mHealth topic.

Region	Micro-Topics Labeled as MHealth Topics
Region 1	IoT Healthcare Systems: Leveraging IoT for secure, efficient health monitoring and early warning systems.
	Remote Health Monitoring: Systems for tracking patient health remotely to optimize care and reduce costs.
	Wireless Passive Sensors: Integration of wearable sensors for unobtrusive physiological monitoring using wireless resistive analog technology.
Region 2	Continuous Glucose Monitoring: Assessing wearable devices for blood glucose management and glycemic trends in diabetes.
	Digital Phenotyping: Utilizing digital data to identify health changes and improve proactive health measures.
	Smartphone Health Diagnostics: Using smartphones for real-time health monitoring through various sensory and imaging technologies.
Region 3	Heart Failure Self-Care: Mobile apps enhancing patient self-management and reducing hospital readmissions for heart failure.
	Perinatal Health Interventions: Evaluating digital and integrative approaches to support maternal health and infant care.
	Chronic Illness Management: Exploring support, self-management, and technology for patients with multiple chronic conditions.
Region 4	mHealth HIV Interventions: Exploring mobile health strategies to enhance HIV treatment adherence among specific populations.
	Mobile Health Interventions: Implementing SMS systems to support maternal and neonatal health.
	Tuberculosis Treatment Adherence: Utilizing mobile interventions to support and improve TB treatment adherence.
Region 5	Digital Mental Health: Innovations in technology enhancing mental health support and interventions for diverse populations.
	mHealth Addiction Support: Utilizing mobile health apps to aid recovery in substance and alcohol use disorders.
	Peer Support Interventions: Certified peers assist mental health self-management through mentorship and digital support tools.
Region 6	Smoking Cessation: Strategies and interventions for helping individuals quit smoking and maintain abstinence.

**Table 5.** Mean of mHealth Publication Ratio by Cohort (After–Before). The second column reports the number of scholars who had at least one publication in both the pretraining [−3, 0) and post-training (0, 3] time windows.

Cohort	Valid N	Before (%)	After (%)	Δ (After–Before)

2015	19	13.6	27.8	+14.1
2016	23	7.5	22.3	+14.8
2017	29	22.3	28.9	+6.6
2018	17	20.0	21.9	+1.9
2019	24	18.2	29.5	+11.3
2021	28	15.8	18.7	+2.9
2022	23	18.2	16.6	−1.6

**Table 6.** Mean of mHealth Publication Ratio by Discipline (After–Before).

Discipline	Valid N	Before (%)	After (%)	Δ (After–Before)

CS/Stats/Engineering/Data Science	48	26.8	35.4	+8.6
Medicine/Nursing	33	11.6	19.1	+7.5
Psychology	59	12.9	20.1	+7.2
Public Health	15	3.6	6.8	+3.2
Others	8	28.1	28.6	+0.4

**Figure A1.**
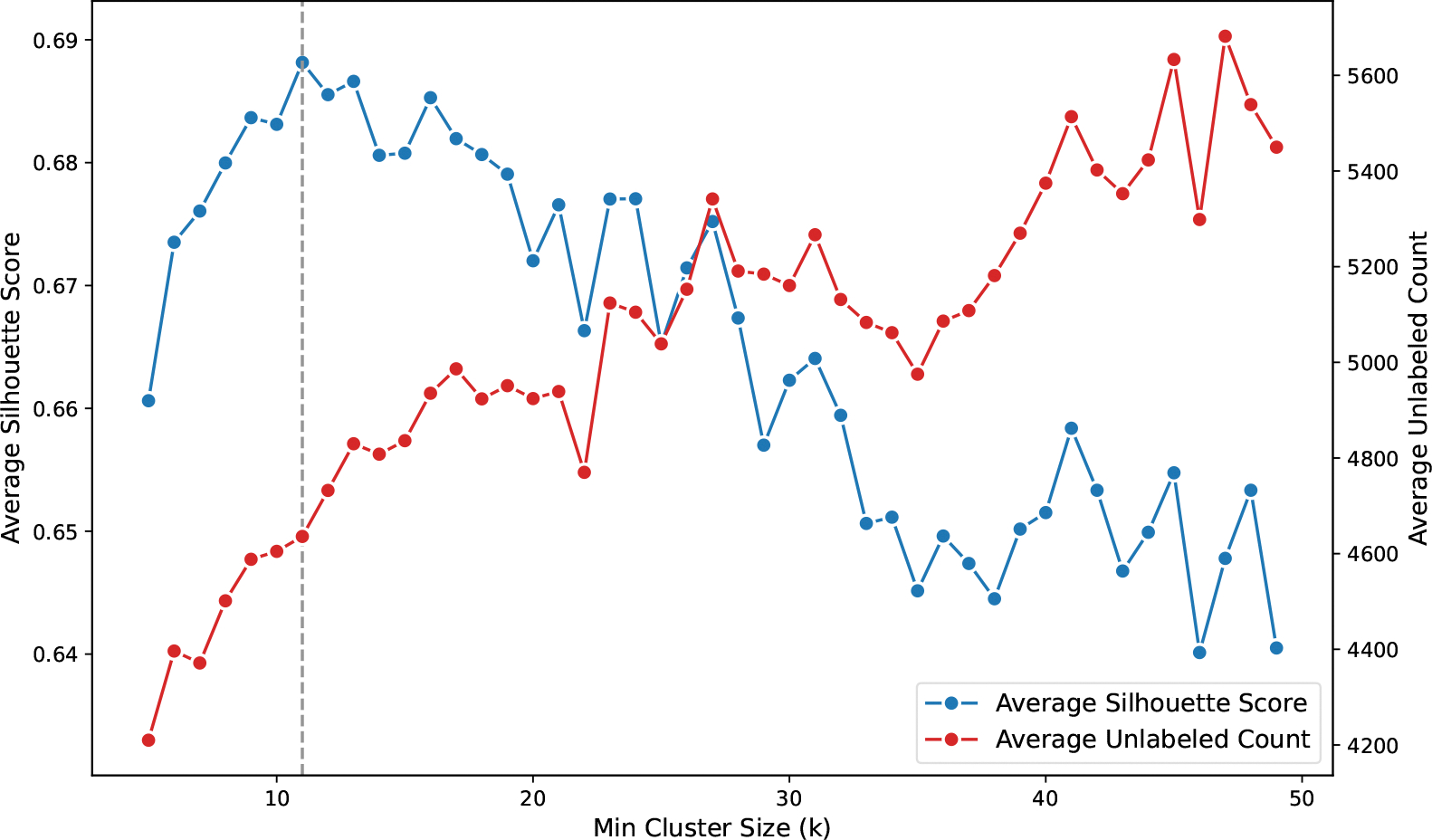
Minimal cluster size determination based on minimum cluster size and average unlabeled counts vs. average Silhouette scores.

## Data Availability

The publication data analyzed in this study are publicly available through PubMed and Google Scholar. Due to Institutional Review Board (IRB) requirements aimed at protecting participant privacy, the scholar demographic data cannot be shared publicly. While these demographic data provide context, they are not essential for replicating the primary analyses or verifying the main conclusions presented in this paper.

## Appendix A. UMAP Configurations

The tuning of hyperparameters is a critical step in the formation of semantic space. For UMAP, we conduct extensive experiments to select optimal hyperparameters, guided by both quantitative metrics and visual inspection of the reduced-dimensional space. We set *min*_*distance* to 0 to compress adjacent points as much as possible, enhancing downstream clustering. The number of neighbors considered during compression is set to 15, as the model’s performance is relatively insensitive to this parameter. To avoid excessive compression, which can result in points collapsing into a straight line, we set the number of components to 5, ensuring a well-distributed representation of the publications in the reduced space.

## Appendix B. HDBSCAN Configurations

For the hyperparameter tuning of HDBSCAN, we focus on two key hyperparameters: *min_cluster_size* and *min_samples*. These control the minimum size of clusters and the density required for a point to be classified as part of a cluster, respectively. To streamline the search for optimal values, we follow a systematic approach by setting both parameters to the same value and testing a range of values from 5 to 50.

We observe a trade-off between the Silhouette score and the min cluster size. Although a large min cluster size is favored for interpretation, it may introduce unsatisfactory Silhouette scores. We plot the average Silhouette scores and average unlabeled counts as a function of the minimum cluster size to determine the optimal cluster sizes, as shown in Figure A1.

## Appendix C. Prompts

**Listing A1.** Prompt for topic label generation.

response = client.chat.completions.create(
model=“gpt-4o“,
messages = [
{
“role”: “system”,
“content”: (
“I am taking research interest analysis of a group of medical scientist accroding to their publications.”
“You are an assistant that generates topic labels and descriptions based on provided keywords and some related documents. The~topic should be commonly used science/medical/biology/technical field, which should not be too concise (Example: mobile health, sexual health, sleep, adhd, cancer care etc.)”
“Your task is to analyze the given keywords and representative documents, then extract a short topic label (no more than 3 words) and a short corresponding sentence as description.”
“The topics must reflect a SINGLE topic instead of a combination of topics.”
)
},
{
“role”: “user”,
“content”: (
“I have a topic described by the following keywords, which might be helpful: {}\n”
“In this topic, I randomly chose 10 documents stored in python list:\n{}\n\n”
“Based on the information above, please extract a short topic label (no more than 3 words) and give a corresponding brief description (in 15 words)in the following format:\n”
“<topic label>: <description>”
).format(keywords, documents)
}
]
)

**Listing A2.** Prompt for unlabeled paper classification.

system_prompt = f“”“You are a helpful assistant specialized in topic classification. You have generated ^~^300 several detailed topic and description according to the publication titles and abstract, while you now need to assign them into below 30 topics, which can be considered as broader and higher level topics.
You have a fixed list of broader classes indexed as follows:
1. Mental Health: Studies mental health issues and their interventions, focusing on emotional regulation, cognitive impairments, and~the diagnosis and treatment of psychological disorders.
2. Medical Care Systems: Analyzes and improves the organization, processes, and^~^efficiency of healthcare services, aiming to optimize patient care experience and enhance the overall effectiveness of healthcare systems.
3. Nutrition & Nursing: Examines the link between diet and health, focusing on nutritional interventions, dietary habits, and~their role in managing health conditions and supporting patient care.
4. Sleep & Fatigue: Studies the factors influencing sleep quality and fatigue management, exploring the long-term effects of sleep disorders, insomnia, and^~^daytime fatigue on physical and mental health, and^~^discusses strategies to improve sleep and reduce fatigue.
5. Chronic Conditions: Explores the management and treatment methods for chronic diseases.
6. Cancer: Specializes in the early diagnosis, treatment methods, care processes, and^~^survivorship support for cancer patients.
7. Clinical Research: Explores the design and implementation of clinical research, including clinical trials for new therapies and drugs, disease screening, and^~^prevention studies. The^~^aim is to support the effectiveness and safety of treatment protocols through evidence-based research, improving clinical practice.
8. Genetic and Cellular Research: Studies the biological processes at the genetic and cellular levels.
9. Infectious Disease: Focuses on the prevention, diagnosis, and^~^vaccine development of infectious diseases and their global health impact.
10. Public Health: Focuses on community and population-level health issues, emphasizing health promotion, the^~^implementation of public health policies, disease prevention, and^~^social health advocacy.
11: Sexuality and Gender: Sexual Behavior, Gender Dynamics etc
12. Brain and Neural Science: Studies the structure, function, and^~^diseases of the brain and nervous system, exploring advances in neurobiology, cognitive function, neurodegenerative diseases (e.g., Alzheimer’s, Parkinson’s), and^~^brain-behavior relationships.
13. Society and Business: Examines the intersection of societal issues and business operations outside the scope of public health. Key areas include social policies, business decisions, economic models, social influence, and^~^business ethics.
14. Technology & Engineering: Covers a wide range of topics in science, technology, engineering, and^~^applied sciences that do not belong to other categories. This includes emerging technologies (e.g., AI, IoT, robotics), engineering design, information systems, and^~^data analysis.
When given a detailed topic’s description and 10 random sampled articles below the topics, choose the index number of the best matching topic from the list.
You [must only return the index number] as a string, no explanation.
“”“
user_prompt = f“”“
Please read the following topic’s description and paper details under the topic, then determine the index number of the broader class this topic belongs to.
You [must only return the index number].
Topic and description: {topic_des}
Articles: {text4emb_list}
“”“

**Listing A3.** Prompt for topic description refinement.

system_prompt = “”“You are an intelligent assistant that specializes in refining and modifying topic descriptions based on given instructions.
Your task is to carefully analyze the provided topic and its description while preserving its original meaning.
Ensure that the newly generated description remains as close as possible to the original version, except^~^for the specific modifications requested. “”“
user_prompt = f“”“
Please analyze the following topic and its description in the format Topic: Description.
If the description contains concrete geographical locations (such as village names, states, provinces, or~countries), remove these place names while ensuring the remaining content remains meaningful and^~^coherent.
Example 1:
Alcohol Use in HIV: Exploring impacts and patterns of alcohol use among HIV patients in Uganda.
Uganda is a concrete country's name, which should be removed. Thus, you	should output:
Alcohol Use in HIV: Exploring impacts and patterns of alcohol use among HIV^~^patients.
Example 2:
Palliative Care Access: Challenges in opioid availability and cancer pain management in low-income countries.
There is no concrete geographic location name. No need to change, just output the original^~^one.
Here is the input topic description: {topic_des}
You should output the modified topic and description strictly in the original format (Topic: Description).
“”“

## Appendix D. Partial Mapping From Meso- to Micro-Topics

Cluster 1:

- Pediatric Asthma Management: Focus on improving techniques, adherence, and care outcomes in children with asthma.
- Sleep Health: Examines the impact of COVID-19 and lifestyle factors on sleep behaviors and quality.
- ICU Delirium: Delirium in ICU patients, its detection, education, environmental factors, and management strategies.

Cluster 2:

- Virtual Reality Assessment: Evaluating cognitive functions using VR technology improves accuracy and ecological validity.
- Functional Brain Networks: Examines connections and dynamics of brain regions under various cognitive conditions.
- Virtual Patient Training: Utilizing virtual characters to enhance clinical skills in interviewing PTSD patients.

Cluster 3:

- Alcohol Use in HIV: Exploring impacts and patterns of alcohol use among HIV patients.
- Injection Drug Use: Investigating risks and transmissions among injection drug users in urban environments.
- HIV Neurocognitive Disorders: Explores cognitive impairments and disorders among HIV-positive individuals on antiretroviral therapy.

Cluster 4:

- Behavioral Weight Loss: Focuses on interventions and strategies to promote weight loss in diverse populations.
- Exercise and Health: Impact of aerobic and resistance exercises on health, fitness, and metabolic outcomes.
- Postmenopausal Health: Examining weight, diet, metabolic factors impacting heart disease and diabetes post-menopause.

Cluster 5:

- Atrial Fibrillation Genetics: Studies identify genetic factors influencing atrial fibrillation risk and related conditions.
- Alzheimer’s Disease Diagnosis: Methods and biomarkers improving Alzheimer’s diagnosis and understanding cognitive decline patterns.
- Pharmacogenomics Prediction: Machine learning predicts drug response in MDD, osteoporosis using genomic, clinical data.

Cluster 6:

- Parasite–Host Interactions: Explores ecological and immunological interactions between parasites and hosts impacting evolution and virulence.
- Pediatric Respiratory Infections: Study of bacterial pneumonia, pertussis, and meningitis impact on children.
- Antibiotic Resistance: Examines prevalence and control measures of methicillin-resistant bacteria in healthcare settings.

Cluster 7:

- ADHD Pediatric Treatment: Examining ADHD treatments in children; focuses on medication, behavioral therapy, and learning interventions.
- Conduct Disorders: Study of behavioral and emotional issues in children relating to oppositional defiant disorder.
- Reaction Time Variability: Examines ADHD-related reaction time variability impacts on cognitive functioning in children and adults.

Cluster 8:

- Smoking Cessation: Strategies and interventions for helping individuals quit smoking and maintain abstinence.
- E-cigarette Use: Examining e-cigarette prevalence, impacts on health, youth usage trends, and product preferences.
- Smoking Cessation: Examines behavioral, psychological factors affecting attempts to quit smoking using ecological momentary assessments.

Cluster 9:

- Emergency Workflow: Investigating workflows in emergency settings to enhance patient care and system efficiency.
- Telemedicine Innovations: Advancements and challenges in telemedicine across various medical specialties post-COVID-19.
- Deliberative Democracy: Evaluating public opinions and decisions through structured group discussions on social policies.

Cluster 10:

- Fault Tolerant Systems: Techniques ensuring system reliability amid faults, including replication, consistency, and Byzantine fault tolerance.
- Dark Silicon Management: Strategies to optimize power and performance in many-core systems under constraints.
- Wireless Sensor Networks: Developing energy-efficient communication protocols for sustainable sensor network operations and extended lifespans.

Cluster 11:

- Substance Use Interventions: Strategies and studies addressing substance use among young adults, focusing on mindfulness and motivations.
- Opioid Crisis: Examines opioid misuse, prescribing biases, and public health approaches to the opioid epidemic.
- Adolescent Substance Use: Examines prevalence and screening of substance use among adolescents in various settings.

Cluster 12:

- Anxiety Disorders: Examination of anxiety disorders, assessment tools, and related risk factors in various populations.
- Emotion Regulation: Investigating strategies and effectiveness in managing emotions for psychological well-being.
- Obsessive-Compulsive Disorder: Study of OCD symptoms, their associations, comorbidities, and potential interventions across settings.

Cluster 13:

- COVID-19 Mental Health: Examines COVID-19’s impact on mental health, distress, protective factors, and public perception.
- Healthcare Worker Burnout: Examines mental health and burnout in healthcare workers during the COVID-19 pandemic.
- Nurse Practitioner Burnout: Examining burnout in nurse practitioners and its impact on primary care quality.

Cluster 14:

- IoT Healthcare Systems: Leveraging IoT for secure, efficient health monitoring and early warning systems.
- Underwater Visible Light Communication: Exploring camera-based localization in underwater visible light communication systems for error reduction.
- Image Processing Technology: Techniques for analyzing and manipulating images using advanced imaging apparatus and methods.

Cluster 15:

- Multiclass Learning: Study of algorithms and methods for efficient multiclass classification and ranking tasks.
- Causal Bandit Analysis: Investigation of algorithms leveraging causal knowledge for improved decision-making under uncertainty.
- Survival Analysis: Analyzing time-to-event data with methods for censored and missing data challenges.

Cluster 16:

- Cardiac Magnetic Resonance: Advanced imaging technique for diagnosing coronary artery disease and cardiac abnormalities.
- Platinum-Group Elements: Study of PGE distribution, formation, and geochemistry in sulfide deposits and rocks.
- Cerebral Venous Thrombosis: Research on predictors, treatment options, and complications of cerebral venous thrombosis.

Cluster 17:

- Medicaid Mental Health: Examines Medicaid’s role and challenges in providing mental health services to various populations.
- Racial Discrimination: Examining racial discrimination’s impact on people’s psychological health and identity development.
- Serious Mental Illness: Challenges and care approaches for older adults with serious mental illnesses.

Cluster 18:

- PTSD and Cardiovascular Risk: Examines PTSD’s link to cardiovascular disease risks in affected individuals.
- PTSD and Substance Use: Examines the treatment of co-occurring PTSD and substance use disorders in adolescents and veterans.
- PTSD Genomics: Analyzing genetic links and risk factors related to post-traumatic stress disorder.

Cluster 19:

- Nursing Home Quality: Evaluates nursing home quality metrics including staffing, resident satisfaction, care outcomes, and costs.
- Hematopoietic Cell Transplantation: Study of diverse outcomes and complications in hematopoietic cell transplant patients.
- PACE Programs: Evaluates care models and outcomes for the frail elderly in all-inclusive health settings.

Cluster 20:

- Child Nutrition Interventions: Examines strategies for sustaining nutrition and physical activity programs for young children.
- Children’s Nutrition: Examines strategies to improve children’s dietary choices in restaurants and school cafeterias.
- Gestational Weight Gain: Examining factors influencing weight changes during pregnancy and postpartum for intervention strategies.

Cluster 21:

- Geriatric Oncology: Tailored cancer care for older adults, focusing on assessments and decision-making.
- Cancer Care Technology: Utilizing technology to assess, monitor, and support care for cancer patients and survivors.
- End-of-life Care: Examines spiritual and personalized support in advanced cancer patients’ end-of-life experiences.

Cluster 22:

- Urban Green Spaces: Study of greenness, its health benefits, and impact on urban residents’ well-being.
- Neighborhood Walkability: Investigates how neighborhood design impacts health, physical activity, and environmental exposure.
- Prenatal Air Pollution: Examines how prenatal air pollution exposure impacts child neurodevelopment, including autism risk.

Cluster 23:

- Ebola Virus Outbreaks: Analysis of health responses and impacts during Ebola virus outbreaks.
- Pediatric Dehydration: Evaluating dehydration in children with diarrhea using clinical and tool-based assessments.
- Emergency Trauma Care: Studying trauma treatment and epidemiological challenges in low- and middle-income regions.

Cluster 24:

- Adrenarche and Puberty: Investigating hormonal changes and their influence on physiology, behavior, and mental health.
- Adolescent Emotional Socialization: Examines parental influence on adolescent emotions and impact on mental health.
- Adolescent Inflammatory Markers: Examining inflammation’s role and psychosocial factors in adolescents’ cardiovascular health.

Cluster 25:

- mHealth HIV Interventions: Exploring mobile health strategies to enhance HIV treatment adherence among specific populations.
- Mobile Health Interventions: Implementing SMS systems to support maternal and neonatal health in Kenya.
- Maternal Health Disparities: Examines disparities in maternal health and infant mortality among racial and ethnic groups.

Cluster 26:

- Micro-fluidic Biosensors: Integration of CMOS technology for advanced biofluid analysis and cell culture monitoring.
- Point-of-Care Diagnostics: Rapid testing technologies for detecting biomarkers and antigens at point-of-care settings.
- Electroporation Techniques: Study of electric pulses for cell membrane permeability and DNA extraction processes.

Cluster 27:

- mHealth Addiction Support: Utilizing mobile health apps to aid recovery in substance and alcohol use disorders.
- Peer Support Interventions: Certified peers assist mental health self-management through mentorship and digital support tools.
- Microrandomized Trials: Experimental design optimizing adaptive mobile health interventions through sequential randomization and engagement strategies

Cluster 28:

- Sexual Minority Health: Examines psychosocial wellbeing, stigma, and identity in gay and bisexual men’s health.
- Youth Slum Challenges: Examines risks of HIV, violence, and exploitation among youth in Kampala slums.
- Adolescent Sexual Behavior: Examines factors and effects of sexual behavior in adolescents, including risk and motivations.

Cluster 29:

- Bladder Cancer Diagnosis: Techniques for enhancing detection and management of bladder cancer and patient outcomes.
- Radical Cystectomy Outcomes: Examines operative techniques, complications, and opioid use in radical cystectomy patients.
- Urology Workforce: Analysis of trends in demographics and gender disparities within the urology workforce.

Cluster 30:

- Fiber-Optic Sensing: Techniques for detecting vibrations and enhancing intrusion detection via fiber-optic technologies.
- Fiber-Optic Sensors: Sensors using fiber optics for precise strain, temperature, and refractive index measurements.
- Graphene-Based Gas Sensing: Advanced gas sensors using graphene for highly sensitive and selective detection.

Cluster 31:

- Cardiac Signal Analysis: Techniques for evaluating ECG and PPG signals to identify cardiac events and health.
- EEG and Sleep: Explores EEG application in sleep-related studies, including deficiency, artifact removal, and sleep stages.
- Speech-Based Depression Detection: Identifying depression signs in adolescents using speech signal analysis and classification techniques.

Cluster 32:

- Agile Project Management: Examines agile methods for enhancing visibility and coordination in project development tasks.
- Startup Financing Contracts: Examination of how know-how and stage-based contracts affect startup valuation and investment.
- Firm Location Decisions: Examines factors influencing firms’ geographical choices, including incentives, agglomeration economies, and home bias.

Cluster 33:

- Political Ethics: Examining ethics and conduct within politics and public perceptions of political integrity.
- Literary Revival: Exploration of cultural history and identity via literary and artistic works.
- Peer Review Acknowledgements: Expressions of gratitude towards individuals who contributed to the peer review process.

Cluster 34:

- Vibrotactile Therapy: Exploring vibration techniques to improve sensory and motor functions post-stroke.
- Grip Mechanics: Study of hand–object interaction and friction effects on grip strength and safety.
- Grip Impairments Post-Stroke: Examines grip force challenges and sensory deficits in stroke survivors’ hands.

Cluster 35

- Kinect Motion Tracking: Employing Microsoft Kinect for real-time motion tracking and assessment in healthcare and rehabilitation.
- Soft Robotic Actuators: Focused on designing and utilizing soft actuators for adaptive and efficient robotic systems.
- Knee Exosuit Rehabilitation: Investigates knee exosuit benefits in gait assistance, rehabilitation, and muscular effort reduction.

Cluster 36:

- Digital Youth Identity: Examining how digital media influences identity development among today’s young people.
- Digital Badges: Exploring the use of digital badges to recognize achievements in student education programs.
- Electrical Engineering Education: Innovative approaches to teaching electrical engineering, emphasizing project-based learning and entrepreneurship.

Cluster 37:

- Maternal Health Empowerment: Exploring health interventions and challenges for mothers of children with disabilities.
- Cerebral Palsy Support: Examining family experiences and technology use for children with cerebral palsy.
- Occupational Therapy Australia: Examines practices, challenges, and advancements in occupational therapy within the Australian context.

Cluster 38:

- Cross-Technology Communication: Efficient data dissemination among IoT using varied protocols like WiFi and Zigbee.
- Blockchain Security: Examines blockchain’s security role in data integrity, voting, IoT, and smart contracts.
- IoT Systems Interoperability: Techniques for integrating heterogeneous IoT devices across platforms and communication standards.

Cluster 39:

- Voice Assistants Privacy: Examines privacy concerns and benefits of voice assistants supporting older adults’ independence.
- Chronic Illness Management: Exploring support, self-management, and technology for patients with multiple chronic conditions.
- Conversational Agents: AI-driven systems enabling interactive, personalized dialogue in health, fitness, and education.

Cluster 40:

- Diabetes in Youth: Examines prediabetes and diabetes diagnosis and awareness among youth and adolescents.
- Continuous Glucose Monitoring: Assessing wearable devices for blood glucose management and glycemic trends in diabetes.
- Continuous Glucose Monitoring: Examines CGM’s role, challenges, and facilitators in managing type 1 and 2 diabetes.

## References

[1] Menken S; Keestra M An Introduction to Interdisciplinary Research: Theory and Practice, 3rd ed.; Amsterdam University Press: Amsterdam, The Netherland, 2016.

[2] Okamura K Interdisciplinarity revisited: Evidence for research impact and dynamism. Palgrave Commun. 2019, 5, 1–9.

[3] Iyawa GE; Herselman M; Botha A Digital health innovation ecosystems: From systematic literature review to conceptual framework. Procedia Comput. Sci. 2016, 100, 244–252.

[4] Istepanian RS Mobile health (m-Health) in retrospect: The known unknowns. Int. J. Environ. Res. Public Health 2022, 19, 3747. 35409431 10.3390/ijerph19073747 PMC8998037

[5] Garai A Seven factors for designing successful mHealth projects. XRDS Crossroads ACM Mag. Stud. 2012, 19, 16–19.

[6] Fortunato S; Bergstrom CT; Börner K; Evans JA; Helbing D; Milojevic S; Petersen AM; Radicchi F; Sinatra R; Uzzi B; et al. Science of science. Science 2018, 359, eaao0185. 29496846 10.1126/science.aao0185 PMC5949209

[7] Porter AL; Rafols I Is science becoming more interdisciplinary? Measuring and mapping six research fields over time. Scientometrics 2009, 81, 719–745.

[8] Wuchty S; Jones BF; Uzzi B The Increasing Dominance of Teams in Production of Knowledge. Science 2007, 316, 1036–1039. 17431139 10.1126/science.1136099

[9] Ho E; Jeon M; Lee M; Luo J; Pfammatter AF; Shetty V; Spring B Fostering interdisciplinary collaboration: A longitudinal social network analysis of the NIH mHealth Training Institutes. J. Clin. Transl. Sci. 2021, 5, e191. 34849265 10.1017/cts.2021.859 PMC8596066

[10] Aboelela SW; Larson E; Bakken S; Carrasquillo O; Formicola A; Glied SA; Haas J; Gebbie KM Defining interdisciplinary research: Conclusions from a critical review of the literature. Health Serv. Res. 2007, 42, 329–346. 17355595 10.1111/j.1475-6773.2006.00621.x PMC1955232

[11] Silva BM; Rodrigues JJ; de la Torre Díez I; López-Coronado M; Saleem K Mobile-health: A review of current state in 2015. J. Biomed. Inform. 2015, 56, 265–272. 26071682 10.1016/j.jbi.2015.06.003

[12] Steinhubl SR; Muse ED; Topol EJ The emerging field of mobile health. Sci. Transl. Med. 2015, 7, 283rv3. 10.1126/scitranslmed.aaa3487 PMC4748838 25877894

[13] Mazzocchi F Scientific research across and beyond disciplines: Challenges and opportunities of interdisciplinarity. EMBO Rep. 2019, 20, e47682. 31040110 10.15252/embr.201947682 PMC6549017

[14] Campbell LM Overcoming obstacles to interdisciplinary research. Conserv. Biol. 2005, 19, 574–577.

[15] Nilsen W; Kumar S; Shar A; Varoquiers C; Wiley T; Riley WT; Pavel M; Atienza AA Advancing the science of mHealth. J. Health Commun. 2012, 17, 5–10. 10.1080/10810730.2012.677394 22548593

[16] Blei DM; Ng AY; Jordan MI Latent dirichlet allocation. J. Mach. Learn. Res. 2003, 3, 993–1022.

[17] Hofmann T Probabilistic latent semantic indexing. In Proceedings of the 22nd Annual International ACM SIGIR Conference on Research and Development in Information Retrieval, Berkeley, CA, USA, 15–19 August 1999; pp. 50–57.

[18] Yau CK; Porter A; Newman N; Suominen A Clustering scientific documents with topic modeling. Scientometrics 2014, 100, 767–786.

[19] Owa DLM Identification of topics from scientific papers through topic modeling. Open J. Appl. Sci. 2021, 10, 541.

[20] Grootendorst M BERTopic: Neural topic modeling with a class-based TF-IDF procedure. arXiv 2022, arXiv:2203.05794.

[21] Pham CM; Hoyle A; Sun S; Resnik P; Iyyer M Topicgpt: A prompt-based topic modeling framework. arXiv 2023, arXiv:2311.01449.

[22] Lee K; Chung Y; Kim JS Research Trends on Metabolic Syndrome in Digital Health Care Using Topic Modeling: Systematic Search of Abstracts. J. Med. Internet Res. 2024, 26, e53873. 39666378 10.2196/53873 PMC11671787

[23] Shan Y; Ji M; Xie W; Lam KY; Chow CY Public trust in artificial intelligence applications in mental health care: Topic modeling analysis. JMIR Hum. Factors 2022, 9, e38799. 36459412 10.2196/38799 PMC9758643

[24] Ahmed A; Aziz S; Khalifa M; Shah U; Hassan A; Abd-Alrazaq A; Househ M Thematic analysis on user reviews for depression and anxiety chatbot apps: Machine learning approach. JMIR Form. Res. 2022, 6, e27654. 35275069 10.2196/27654 PMC8956988

[25] McGrath ER; Bacso DR; Andrews JG; Rice SA Intentional interprofessional leadership in maternal and child health. Leadersh. Health Serv. 2019, 32, 212–225. 10.1108/LHS-04-2018-0026 30945599

[26] Knapke JM; Tsevat J; Succop PA; Djawe K; Kuhnell P; Haynes EN Publication track records as a metric of clinical research training effectiveness. Clin. Transl. Sci. 2013, 6, 458–462. 24330690 10.1111/cts.12089 PMC3869033

[27] Knapke JM; Haynes EN; Kuhnell P; Tsevat J NIH grant awards as a metric of clinical and translational research training effectiveness. Clin. Transl. Sci. 2015, 8, 52–56. 25377275 10.1111/cts.12232 PMC4329077

[28] Tumin D; Brewer KL; Cummings DM; Keene KL; Campbell KM Estimating clinical research project duration from idea to publication. J. Investig. Med. 2022, 70, 108–109. 10.1136/jim-2021-001915 PMC8127282 33990370

[29] Alcaraz C; Morais S Citations: Results differ by database. Nature 2012, 483, 36–36. 10.1038/483036d 22382969

[30] Cholewiak SA; Ipeirotis P; Silva V; Kannawadi A SCHOLARLY: Simple Access to Google Scholar Authors and Citation Using Python. 2021. Available online: https://github.com/scholarly-python-package/scholarly (accessed on 31 May 2025).

[31] Abuhay TM; Nigatie YG; Kovalchuk SV Towards predicting trend of scientific research topics using topic modeling. Procedia Comput. Sci. 2018, 136, 304–310.

[32] Xiong H; Cheng Y; Zhao W; Liu J Analyzing scientific research topics in manufacturing field using a topic model. Comput. Ind. Eng. 2019, 135, 333–347.

[33] Zhao WX; Zhou K; Li J; Tang T; Wang X; Hou Y; Min Y; Zhang B; Zhang J; Dong Z; A survey of large language models. arXiv 2023, arXiv:2303.18223.

[34] Reuter A; Thielmann A; Weisser C; Fischer S; Säfken B Gptopic: Dynamic and interactive topic representations. arXiv 2024, arXiv:2403.03628.

[35] Park K; Choe YJ; Veitch V The linear representation hypothesis and the geometry of large language models. arXiv 2023, arXiv:2311.03658.

[36] McInnes L; Healy J; Melville J Umap: Uniform manifold approximation and projection for dimension reduction. arXiv 2018, arXiv:1802.03426.

[37] Donoho D High-Dimensional Data Analysis: The Curses and Blessings of Dimensionality; American Mathematical Society: Providence, RI, USA, 2000; pp. 1–32.

[38] McInnes L; Healy J; Astels S hdbscan: Hierarchical density based clustering. J. Open Source Softw. 2017, 2, 205.

[39] Shahapure KR; Nicholas C Cluster quality analysis using silhouette score. In Proceedings of the 2020 IEEE 7th International Conference on Data Science and Advanced Analytics (DSAA), Sydney, NSW, Australia, 6–9 October 2020; pp. 747–748.

