# Supplementary material for: Mapping the mHealth Nexus: A Semantic Analysis of mHealth Scholars’ Research Propensities Following an Interdisciplinary Training Institute
Source: Appl Sci (Basel). Author manuscript; Available in PMC 2025 Dec 24. (PMC12724682; doi:10.3390/app15116252)
Supplement: Supplementary Material [file NIHMS2124663-supplement-Supplementary_Material.pdf]

Supplementary Materials

Full Mapping From General- to Micro-topics

Mental Health

Eating Disorders Research: Investigation of eating disorder symptoms, interventions, and impacts across various populations. (topic size: 202)

ADHD Pediatric Treatment: Examining ADHD treatments in children; focuses on medication, behavioral therapy, and learning interventions. (topic size: 103)

Substance Use Interventions: Strategies and studies addressing substance use among young adults, focusing on mindfulness and motivations. (topic size: 96)

Anxiety Disorders: Examination of anxiety disorders, assessment tools, and related risk factors in various populations. (topic size: 94)

COVID-19 Mental Health: Examines COVID-19's impact on mental health, distress, protective factors, and public perception. (topic size: 85)

Smoking Cessation: Examines behavioral, psychological factors affecting attempts to quit smoking using ecological momentary assessments. (topic size: 80)

Digital Mental Health: Innovations in technology enhancing mental health support and interventions for diverse populations. (topic size: 80)

Medicaid Mental Health: Examines Medicaid's role and challenges in providing mental health services to various populations. (topic size: 78)

Racial Discrimination: Examining racial discrimination's impact on psychological

health and identity development. (topic size: 70)

Emotion Regulation: Investigating strategies and effectiveness in managing emotions for psychological well-being. (topic size: 64)

College Drinking Behaviors: Examines alcohol use, consequences, and interventions among college students with varied motives. (topic size: 62)

mHealth Addiction Support: Utilizing mobile health apps to aid recovery in substance and alcohol use disorders. (topic size: 58)

Peer Support Interventions: Certified peers assist mental health self-management through mentorship and digital support tools. (topic size: 57)

Healthcare Worker Burnout: Examines mental health and burnout in healthcare workers during the COVID-19 pandemic. (topic size: 53)

Depression Screening: Evaluating and managing depressive symptoms through structured assessments in diverse healthcare settings. (topic size: 49)

HIV Neurocognitive Disorders: Explores cognitive impairments and disorders among HIV-positive individuals on antiretroviral therapy. (topic size: 45)

Mental Health Prediction: Using data and learning models to predict mental health and wellbeing outcomes. (topic size: 42)

Adolescent Substance Use: Examines prevalence and screening of substance use among adolescents in various settings. (topic size: 39)

Obsessive-Compulsive Disorder: Study of OCD symptoms, their associations, comorbidities, and potential interventions across settings. (topic size: 39)

Hoarding Disorder: Examines hoarding symptoms, comorbidities, and psychological aspects within diverse populations and disorders. (topic size: 38)

Anxiety Treatment Therapies: Study of CBT and ACT effectiveness for treating social phobia and anxiety disorders. (topic size: 36)

Mindfulness and Smoking: Exploring mindfulness approaches to aid smoking cessation, targeting diverse and underserved populations. (topic size: 34)

Suicidal Behavior Adolescents: Examines risk factors, coping, and interventions for youth in suicide-related crises. (topic size: 33)

Adolescent Emotional Socialization: Examines parental influence on adolescent emotions and impact on mental health. (topic size: 33)

PTSD and Substance Use: Examines the treatment of co-occurring PTSD and substance use disorders in adolescents and veterans. (topic size: 30)

Speech-Based Depression Detection: Identifying depression signs in adolescents using speech signal analysis and classification techniques. (topic size: 30)

Serious Mental Illness: Challenges and care approaches for older adults with serious mental illnesses. (topic size: 30)

Cerebral Palsy Support: Examining family experiences and technology use for children with cerebral palsy. (topic size: 28)

Virtual Patient Training: Utilizing virtual characters to enhance clinical skills in interviewing PTSD patients. (topic size: 27)

Interpersonal Suicide Theory: Examines factors like burdensomeness and belongingness in suicidal ideation and behavior. (topic size: 24)

Mobile Sensing Mental Health: Studying depression and anxiety in students using smartphones and passive data collection. (topic size: 23)

Autism Emotion: Examining unique emotional processing impairments in children with Autism Spectrum Disorder (ASD). (topic size: 23)

Dementia Caregiving: Exploring family caregivers' stress, interventions, and support for dementia-related challenges. (topic size: 23)

Adolescent Inflammatory Markers: Examining inflammation's role and psychosocial factors in adolescents' cardiovascular health. (topic size: 23)

Heart Failure Cognition: Examines cognitive deficits and interventions in older adults with heart failure. (topic size: 22)

Alcohol Use Motives: Examines reasons for alcohol consumption, focusing on mood states and expectancy effects. (topic size: 21)

Schizophrenia and Oxytocin: Investigating oxytocin's potential to modulate cognitive symptoms and social behavior in schizophrenia. (topic size: 21)

Virtual Reality Therapy: Using VR for exposure therapy to treat PTSD in military personnel. (topic size: 20)

Parental Illness Uncertainty: Examines effects of parents' uncertainty on psychological distress in chronic illness contexts. (topic size: 20)

Alcohol-Induced Blackouts: Exploring memory impairment from drinking, focusing on college students' experiences and perceptions. (topic size: 18)

Conduct Disorders: Study of behavioral and emotional issues in children relating to oppositional defiant disorder. (topic size: 18)

Psychotic Disorders Adherence: Exploring medication adherence challenges and interventions in psychotic-spectrum disorder treatment. (topic size: 17)

Psychological Health and Longevity: Examines how optimism affects heart health and reduces mortality risks. (topic size: 17)

Social Networking Impact: Examines individual differences and mental health outcomes relating to social networking site use. (topic size: 17)

Digital Geriatric Care: Enhancing older adult mental health with digital, mobile interventions and research innovations. (topic size: 16)

Emotion-Related Impulsivity: Examining how impulsivity tied to emotion predicts internalizing symptoms and psychopathology. (topic size: 16)

Firefighter Suicide: Investigating suicide patterns and risk factors among firefighter

populations and related demographics. (topic size: 16)

Reaction Time Variability: Examines ADHD-related reaction time variability impacts on cognitive functioning in children and adults. (topic size: 15)

Sluggish Cognitive Tempo: Examining SCT's unique psychological traits, academic impacts, and social difficulties in ADHD. (topic size: 15)

Digital Mood Biomarkers: Analyzing smartphone keyboard dynamics to assess mood disorders through metadata and keystroke patterns. (topic size: 15)

Mindfulness-Based Interventions: Programs using mindfulness to reduce stress, enhance cognitive function, and improve mental health. (topic size: 14)

AIDS Orphans: Examines psychosocial impact and well-being of children orphaned by AIDS. (topic size: 12)

Autobiographical Memory: Examines memory's role in depression, focusing on overgeneral memory and psychological assessments. (topic size: 12)

Adolescent Disaster Mental Health: Examines PTSD, depression, and substance use in youths post-tornado exposure. (topic size: 12)

Sexuality & Gender

Gender-Affirming Care: Examines medical, psychosocial aspects and fertility preservation in transgender and gender-diverse youth. (topic size: 119)

Sexual Minority Health: Examines health disparities and risk factors among sexual and gender minority populations. (topic size: 73)

Sexual Minority Health: Examines psychosocial wellbeing, stigma, and identity in gay and bisexual men's health. (topic size: 58)

Adolescent Reproductive Health: Examines reproductive care, contraception, and sexual health issues among adolescents in emergency settings. (topic size: 54)

Adolescent Sexual Behavior: Examines factors and effects of sexual behavior in adolescents, including risk and motivations. (topic size: 46)

Sexual Violence Perpetration: Examines factors influencing sexual violence perpetration, focusing on alcohol, attitudes, and media influences. (topic size: 44)

Digital Youth Identity: Examining how digital media influences identity development among today's young people. (topic size: 39)

Female Sex Workers: Examining health, risks, and experiences of female sex workers. (topic size: 38)

Genital Fistula Recovery: Exploration of women's recovery and reintegration post-genital fistula surgery. (topic size: 29)

Intimate Partner Violence: Examining factors predicting IPV and sexual risk behaviors among young men. (topic size: 19)

Sexual Health Texting: Exploring text message interventions promoting sexual health among teens and young adults. (topic size: 19)

LGBTQ+ Tobacco Use: Examines tobacco use disparities and health communication in LGBTQ+ communities. (topic size: 14)

Sex Trafficking: Examines challenges interventions for trafficked women, focusing on empowerment and rehabilitation. (topic size: 12)

Technology & Engineering

Fault Tolerant Systems: Techniques ensuring system reliability amid faults, including replication, consistency, and Byzantine fault tolerance. (topic size: 98)

IoT Healthcare Systems: Leveraging IoT for secure, efficient health monitoring and early warning systems. (topic size: 85)

Multiclass Learning: Study of algorithms and methods for efficient multiclass classification and ranking tasks. (topic size: 83)

Dark Silicon Management: Strategies to optimize power and performance in many-core systems under constraints. (topic size: 77)

Platinum-Group Elements: Study of PGE distribution, formation, and geochemistry in sulfide deposits and rocks. (topic size: 75)

Underwater Visible Light Communication: Exploring camera-based localization in underwater visible light communication systems for error reduction. (topic size: 72)

Image Processing Technology: Techniques for analyzing and manipulating images using advanced imaging apparatus and methods. (topic size: 60)

Microfluidic Biosensors: Integration of CMOS technology for advanced biofluid analysis and cell culture monitoring. (topic size: 59)

Fiber Optic Sensing: Techniques for detecting vibrations and enhancing intrusion detection via fiber-optic technologies. (topic size: 54)

Cardiac Signal Analysis: Techniques for evaluating ECG and PPG signals to identify cardiac events and health. (topic size: 53)

Agile Project Management: Examines agile methods for enhancing visibility and coordination in project development tasks. (topic size: 50)

Fiber-Optic Sensors: Sensors using fiber optics for precise strain, temperature, and refractive index measurements. (topic size: 50)

Technical Committees: Overview of roles and members in conference technical program committees across institutions. (topic size: 48)

Wireless Sensor Networks: Developing energy-efficient communication protocols for sustainable sensor network operations and extended lifespans. (topic size: 41)

Causal Bandit Analysis: Investigation of algorithms leveraging causal knowledge for improved decision-making under uncertainty. (topic size: 41)

Land Use Modeling: Analyzing land cover changes using satellite data and machine learning techniques. (topic size: 41)

Biometric Authentication: Enhancing user security on devices using behavioral and biometric authentication methods. (topic size: 39)

Self-Tracking Visualization: Techniques to use visual data exploration for enhancing personal reflection and insights. (topic size: 38)

Graphene-Based Gas Sensing: Advanced gas sensors using graphene for highly sensitive and selective detection. (topic size: 36)

Wireless Passive Sensors: Integration of wearable sensors for unobtrusive physiological monitoring using wireless resistive analog technology. (topic size: 34)

ACL Tissue Engineering: Developing scaffolds to enhance ligament repair using biomaterials for anterior cruciate ligament. (topic size: 34)

3D NoC Architectures: Examines advanced design, thermal challenges, and optimization in three-dimensional network-on-chip systems. (topic size: 31)

Indoor Localization: Techniques and technology for accurately determining positions within indoor environments using sensors and signals. (topic size: 31)

Grip Mechanics: Study of hand-object interaction and friction effects on grip strength and safety. (topic size: 31)

Bayesian Inference Models: Advanced statistical methods for analyzing complex data using Bayesian approaches and Monte Carlo simulations. (topic size: 31)

Wearable Activity Monitoring: Analyzing and validating physical activity using wearable and sensor-based technologies. (topic size: 31)

Flexible Dry Electrodes: Innovation in long-duration biocompatible ECG/EEG monitoring using printed and nanotechnology electrodes. (topic size: 30)

Text Entry Techniques: Exploration of novel methods for efficient and accessible text entry on digital devices. (topic size: 28)

Cross-Technology Communication: Efficient data dissemination among IoT using varied protocols like WiFi and Zigbee. (topic size: 28)

Air Quality Monitoring: Development of systems for monitoring air contaminants using sensors in various settings. (topic size: 27)

Coronary Imaging AI: Utilizing AI in coronary imaging enhances disease diagnosis, risk prediction, and plaque analysis. (topic size: 27)

Voice Assistants Privacy: Examines privacy concerns and benefits of voice assistants supporting older adults' independence. (topic size: 27)

Aerial Robotics Manipulation: Develops autonomous grasping systems for drones using soft robotic grasper technology and control algorithms. (topic size: 24)

Blockchain Security: Examines blockchain's security role in data integrity, voting, IoT, and smart contracts. (topic size: 21)

Soft Robotic Actuators: Focused on designing and utilizing soft actuators for adaptive and efficient robotic systems. (topic size: 21)

Digital Badges: Exploring the use of digital badges to recognize achievements in student education programs. (topic size: 21)

Sustainable Microgrids: Design and management of energy-sharing microgrids to reduce residential electricity costs. (topic size: 21)

Adaptive Control Systems: Study of control policies optimizing performance in un-

certain linear dynamical systems using adaptive algorithms. (topic size: 20)

Electrical Engineering Education: Innovative approaches to teaching electrical engineering, emphasizing project-based learning and entrepreneurship. (topic size: 20)

Wearable EEG Devices: Innovative EEG designs eliminate DRL circuit for improved neurophysiological data acquisition and analysis. (topic size: 19)

Intelligent Electronic Devices: Focus on advancements and innovations in electronic engineering and interdisciplinary system solutions. (topic size: 19)

Epitaxial Thin Films: Study of electronic transport and structural properties in epitaxial C60 thin films. (topic size: 19)

Autism Behavior Monitoring: Using wearable tech and machine learning to track/manage behaviors in children with autism. (topic size: 18)

IoT Systems Interoperability: Techniques for integrating heterogeneous IoT devices across platforms and communication standards. (topic size: 18)

Conversational Agents: AI-driven systems enabling interactive, personalized dialogue in health, fitness, and education. (topic size: 18)

Smartphone Health Diagnostics: Using smartphones for real-time health monitoring through various sensory and imaging technologies. (topic size: 17)

Fiber Optic Sensors: High-frequency laser pulses enhance long-period fiber gratings for torsion and strain sensing. (topic size: 17)

Network-on-Chip Architectures: Focus on reconfigurable FIFOs and fault-tolerant virtual channel management for improved performance. (topic size: 16)

Collocated Mobile Interactions: Exploring synchronous photo sharing and collaborative activities among proximal mobile users. (topic size: 16)

Electric Vehicle Energy Networks: Study of EVs' roles in energy sharing, distribution, and scheduling systems. (topic size: 16)

Plant Phytohormone Sensors: Real-time in-situ monitoring of phytohormones aids crop stress detection and productivity improvement. (topic size: 15)

Exchangeable Network Models: Study of statistical models on relationally invariant structured interactions in network data. (topic size: 15)

Wearable Health Devices: Exploring consumer wearables for accurate health and fitness monitoring and standardization. (topic size: 15)

Federated Privacy Learning: Techniques for secure, privacy-preserving learning in mobile health using federated approaches. (topic size: 14)

Affective Computing: Study of AI systems understanding, mimicking human emotions for improved human-technology interaction. (topic size: 13)

Pain Monitoring Systems: Automated systems using physiological and behavioral data for real-time pain assessment. (topic size: 13)

Brain-Computer Interfaces: Technologies aiding communication, rehabilitation, and monitoring through brain activity analysis and control systems. (topic size: 13)

JITAIs in Addiction: Mobile interventions provide adaptive support for addiction recovery using just-in-time strategies. (topic size: 12)

Self-Aware Systems: Systems with computational self-awareness for enhanced autonomy, adaptability, and performance in dynamic environments. (topic size: 12)

Human-Robot Interaction: Analyzing empathy, trust, and intention estimation in collaborative human-robot environments. (topic size: 11)

Sleep & Fatigue

Sleep Health: Examines the impact of COVID-19 and lifestyle factors on sleep behaviors and quality. (topic size: 83)

Sleep and Cardiovascular Health: Examining sleep's role and patterns in cardiovascular and cardiometabolic disease risk. (topic size: 77)

EEG and Sleep: Explores EEG application in sleep-related studies, including deficiency, artifact removal, and sleep stages. (topic size: 45)

Cancer

Geriatric Oncology: Tailored cancer care for older adults, focusing on assessments and decision-making. (topic size: 74)

Cancer Care Technology: Utilizing technology to assess, monitor, and support care for cancer patients and survivors. (topic size: 62)

End-of-life Care: Examines spiritual and personalized support in advanced cancer patients' end-of-life experiences. (topic size: 61)

Bladder Cancer Diagnosis: Techniques for enhancing detection and management of bladder cancer and patient outcomes. (topic size: 56)

Cancer Survivorship Fatigue: Investigating methods to manage fatigue and improve quality for cancer survivors. (topic size: 42)

Radical Cystectomy Outcomes: Examines operative techniques, complications, and opioid use in radical cystectomy patients. (topic size: 41)

PD-L1 Inhibition: Avelumab showed promise in combination therapies for recurrent cancers targeting PD-L1. (topic size: 37)

Cancer Journey Tools: Developing technologies to support patient needs across cancer diagnosis, treatment, and survivorship. (topic size: 26)

Ovarian Cancer Management: Focuses on chemotherapy and surgical strategies enhancing survival in ovarian cancer patients. (topic size: 26)

Prognostic Awareness: Understanding cancer prognosis to improve patient-caregiver communication and healthcare decisions. (topic size: 26)

Cancer Disparities: Examines racial and socio-economic disparities in cancer diagnosis, treatment, and survival outcomes. (topic size: 24)

Diet and Cancer: Examining dietary influences on cancer risk, particularly related to carbohydrate and sugar intake. (topic size: 23)

AML Geriatric Treatment: Challenges and strategies for treating acute myeloid leukemia in older adults. (topic size: 23)

Cancer Survivorship: Examines mental health and existential challenges in adult cancer survivors across ages. (topic size: 20)

Light Pollution Health: Examines effects of nighttime light exposure on cancer risk and circadian disruption. (topic size: 17)

Expressive Coping in Cancer: Exploring emotional expression and coping strategies in breast cancer patients to improve well-being. (topic size: 17)

Remote Patient Monitoring: Analyzing electronic monitoring systems for oncology patient symptoms and treatment response. (topic size: 17)

Hematologic Malignancies Care: Studies focus on improving care and communication for older AML and MDS patients. (topic size: 16)

Palliative Care Access: Challenges in opioid availability and cancer pain management in low-income countries. (topic size: 15)

Ovarian Cancer Survivorship: Enhancing sexual function and communication in ovarian cancer survivors through interventions. (topic size: 13)

Prostate Cancer Outcomes: Investigating quality of life and treatment effects in prostate cancer patients. (topic size: 12)

Shared Decision-Making: Enhancing ovarian cancer treatment by integrating patient preferences on chemotherapy side effects. (topic size: 12)

Chronic Conditions

Pediatric Asthma Management: Focus on improving techniques, adherence, and care outcomes in children with asthma. (topic size: 198)

PTSD and Cardiovascular Risk: Examines PTSD's link to cardiovascular disease risks in affected individuals. (topic size: 78)

Sickle Cell Management: Strategies and challenges in treatment, adherence, and health outcomes for sickle cell disease. (topic size: 76)

ICU Delirium: Delirium in ICU patients, its detection, education, environmental factors, and management strategies. (topic size: 67)

COPD Readmission: Strategies and impacts of reducing hospital readmissions in chronic obstructive pulmonary disease patients. (topic size: 38)

Heart Failure Self-Care: Mobile apps enhancing patient self-management and reducing hospital readmissions for heart failure. (topic size: 37)

Coronary Endothelial Dysfunction: Study of impaired coronary blood vessel function related to cardiovascular risks. (topic size: 36)

Diabetic Myocardial Infarction: Investigates impacts and outcomes of MI in Type 2 diabetic inpatients. (topic size: 29)

Chronic Illness Management: Exploring support, self-management, and technology for patients with multiple chronic conditions. (topic size: 27)

Heart Failure Monitoring: Advanced techniques and devices are used for predicting and managing heart failure events. (topic size: 26)

Diabetes in Youth: Examines prediabetes and diabetes diagnosis and awareness among youth and adolescents. (topic size: 23)

Continuous Glucose Monitoring: Examines CGM's role, challenges, and facilitators in managing type 1 and 2 diabetes. (topic size: 21)

Continuous Glucose Monitoring: Assessing wearable devices for blood glucose management and glycemic trends in diabetes. (topic size: 21)

Pulmonary Disease Monitoring: Techniques for assessing lung conditions using audio-sensing and smartphone-based systems. (topic size: 21)

Iron Chelation Therapy: Investigating adherence and efficacy of treatments for transfusional iron overload in thalassemia. (topic size: 19)

Grip Impairments Post-Stroke: Examines grip force challenges and sensory deficits in stroke survivors' hands. (topic size: 18)

Bronchopulmonary Dysplasia: Investigating causes and treatments of BPD in extremely premature infants. (topic size: 17)

Continuous Glucose Monitoring: Examines hypoglycemia self-management for Type 1 Diabetes using continuous monitoring systems. (topic size: 17)

Peripheral Neuropathy: Examines symptoms, causes, and impacts of peripheral neuropathy and its quality-of-life effects. (topic size: 15)

Gestational Diabetes Treatment: Trends and impacts of glyburide versus insulin in gestational diabetes management. (topic size: 15)

Chronic Total Occlusion PCI: Investigating outcomes, techniques, and complications of percutaneous coronary interventions in CTO cases. (topic size: 15)

Frailty Assessment Tools: Evaluating tools for predicting health outcomes in frail and prefrail older populations. (topic size: 13)

Coffee and Endothelial Function: Investigates coffee consumption's effects on vascular endothelial health and cardiovascular function. (topic size: 13)

Kidney Disease Management: Investigation of treatments and outcomes in chronic and acute kidney disease contexts. (topic size: 13)

COPD and Sleep: Examining the impact of sleep disorders on COPD outcomes and hospital readmissions. (topic size: 12)

Clinical Research

Cardiac Magnetic Resonance: Advanced imaging technique for diagnosing coronary artery disease and cardiac abnormalities. (topic size: 82)

Hematopoietic Cell Transplantation: Study of diverse outcomes and complications in hematopoietic cell transplant patients. (topic size: 71)

Point-of-Care Diagnostics: Rapid testing technologies for detecting biomarkers and antigens at point-of-care settings. (topic size: 39)

Aortic Valve Intervention: Evaluation of TAVI's clinical outcomes compared to surgical valve replacement procedures. (topic size: 36)

Survival Analysis: Analyzing time-to-event data with methods for censored and missing data challenges. (topic size: 35)

Digital Phenotyping: Utilizing digital data to identify health changes and improve proactive health measures. (topic size: 32)

Dual Antiplatelet Therapy: Evaluation of DAPT duration and efficacy in stent treatment and associated risks. (topic size: 31)

Causal Mediation Analysis: Examines how intermediate factors mediate effects between exposure and outcome. (topic size: 29)

Microrandomized Trials: Experimental design optimizing adaptive mobile health interventions through sequential randomization and engagement strategies. (topic size: 22)

Cardiovascular Risk Assessment: Evaluating cardiovascular risk prediction models for diverse populations and patient data. (topic size: 15)

ACL Reconstruction Prediction: Using machine learning to predict outcomes and revisions in ACL surgeries. (topic size: 14)

Stimuli-Responsive Drug Delivery: Chitosan microbeads with nanoparticles enhance controlled drug release via external stimuli. (topic size: 12)

Publication Corrections: The focus on correcting errors in scientific articles to maintain accurate scholarship. (topic size: 12)

Principal Stratification: Investigating surrogate endpoints and causal estimands in vaccine trials using statistical methods. (topic size: 11)

Genetic & Cellular Research

Atrial Fibrillation Genetics: Studies identify genetic factors influencing atrial fibrillation risk and related conditions. (topic size: 133)

Pharmacogenomics Prediction: Machine learning predicts drug response in MDD, osteoporosis using genomic, clinical data. (topic size: 42)

Electroporation Techniques: Study of electric pulses for cell membrane permeability and DNA extraction processes. (topic size: 34)

Protein Function Prediction: Computational methods for predicting protein functions based on sequence-derived properties. (topic size: 31)

Alzheimer's Genetic Variants: Investigates genetic variants related to Alzheimer's, using whole-genome and exome sequencing techniques. (topic size: 28)

DNA Adducts: The study of DNA modifications by carcinogens using chromatography and postlabeling techniques. (topic size: 24)

Lentiviral Integration: Study lentiviral vector integration in gene therapy for conditions like -thalassemia and HIV. (topic size: 24)

Cardiac Conduction Genetics: Study of genetic variants affecting cardiac conduction and electrocardiographic intervals in populations. (topic size: 20)

PTSD Genomics: Analyzing genetic links and risk factors related to post-traumatic stress disorder. (topic size: 20)

Statistical Gene Testing: Methods enhancing power and accuracy in high-dimensional genetic data analysis. (topic size: 17)

Protein Self-Assembly: Study of proteins forming complex structures through hierarchical self-assembly principles and applications. (topic size: 15)

Gene Expression Aging: Investigating gene expression's role in determining biologic aging versus chronological aging. (topic size: 14)

Histamine Pathway Genetics: Examining genetic variations in the histamine pathway related to asthma and allergies. (topic size: 12)

Infectious Disease

Alcohol Use in HIV: Exploring impacts and patterns of alcohol use among HIV patients. (topic size: 159)

Parasite-Host Interactions: Explores ecological and immunological interactions between parasites and hosts impacting evolution and virulence. (topic size: 120)

Injection Drug Use: Investigating risks and transmissions among injection drug users in urban environments. (topic size: 83)

Ebola Virus Outbreaks: Analysis of health responses and impacts during Ebola virus outbreaks. (topic size: 64)

Pediatric Dehydration: Evaluating dehydration in children with diarrhea using clinical and tool-based assessments. (topic size: 61)

HIV Pre-Exposure Prophylaxis: Focus on PrEP usage and uptake among gay and bisexual men for HIV prevention. (topic size: 60)

mHealth HIV Interventions: Exploring mobile health strategies to enhance HIV treatment adherence among specific populations. (topic size: 60)

Pediatric Respiratory Infections: Study of bacterial pneumonia, pertussis, and meningitis impact on children. (topic size: 54)

Antibiotic Resistance: Examines prevalence and control measures of methicillin-resistant bacteria in healthcare settings. (topic size: 47)

Rotavirus Surveillance: Monitoring rotavirus infection patterns in children to inform prevention and vaccination strategies. (topic size: 46)

HIV Testing Strategies: Exploring HIV self-testing and prevention tactics among gay and bisexual men. (topic size: 45)

HIV Medication Adherence: Enhancing antiretroviral adherence using acceptance-based and novel assessment methods for HIV treatment. (topic size: 30)

Tuberculosis Treatment Adherence: Utilizing mobile interventions to support and improve TB treatment adherence. (topic size: 26)

Convalescent Plasma Therapy: Evaluating plasma efficacy in preventing COVID-19 infection through clinical trials. (topic size: 26)

Rapid Antigen Testing: Evaluating rapid antigen tests for COVID-19 and influenza infection detection effectiveness. (topic size: 22)

LAMP Assay Diagnostics: Rapid detection method for bacterial meningitis pathogens using loop-mediated isothermal amplification. (topic size: 20)

HPV Vaccination: Investigating factors influencing HPV vaccine uptake and hesitancy among diverse populations. (topic size: 19)

HIV Cure Research: Investigating strategies and perceptions of youth in eliminating HIV without lifelong ART. (topic size: 18)

Post-Acute Sequelae (PASC): Studying symptoms, impacts, and care models for COVID-19 survivors facing long-term effects. (topic size: 17)

HIV PrEP Access: Examines policies and strategies increasing access to HIV pre-exposure prophylaxis in diverse settings. (topic size: 15)

Voluntary Medical Circumcision: Utilizing VMMC for HIV prevention among male adolescents. (topic size: 14)

Pharmacy-Based Vaccination: Examines perceptions and strategies for vaccine delivery by pharmacists in urban areas. (topic size: 13)

Public Health

Smoking Cessation: Strategies and interventions for helping individuals quit smoking and maintain abstinence. (topic size: 101)

E-cigarette Use: Examining e-cigarette prevalence, impacts on health, youth usage trends, and product preferences. (topic size: 86)

Opioid Crisis: Examines opioid misuse, prescribing biases, and public health approaches to the opioid epidemic. (topic size: 78)

Urban Green Spaces: Study of greenness, its health benefits, and impact on urban residents' well-being. (topic size: 65)

Neighborhood Walkability: Investigates how neighborhood design impacts health, physical activity, and environmental exposure. (topic size: 53)

Prenatal Air Pollution: Examines how prenatal air pollution exposure impacts child neurodevelopment, including autism risk. (topic size: 49)

Youth Slum Challenges: Examines risks of HIV, violence, and exploitation among youth in slums. (topic size: 49)

Particulate Matter Exposure: Examining health effects of long-term exposure to fine particulate matter pollutants. (topic size: 35)

Youth Violence: Examines dating and peer violence, victimization, and perpetration among urban youth populations. (topic size: 31)

Cigarette Smoking: Examines smoking practices and associated psychosocial factors among adolescents and college students. (topic size: 31)

Environmental Noise Exposure: Investigates aircraft noise effects on sleep and health in large cohorts. (topic size: 31)

Maternal Health Disparities: Examines disparities in maternal health and infant mortality among racial and ethnic groups. (topic size: 30)

Heated Tobacco Products: Evaluation of marketing, regulation, and usage of IQOS. (topic size: 29)

Health Disparities: Explores systemic health inequities exacerbated by the COVID-19 pandemic and advocacy strategies. (topic size: 29)

Maternal Health Empowerment: Exploring health interventions and challenges for mothers of children with disabilities. (topic size: 28)

Indigenous Maternal Smoking: Addressing smoking cessation among pregnant women with culturally tailored interventions. (topic size: 26)

Youth Smoking Cessation: Research focused on effective interventions and strategies for reducing youth smoking rates. (topic size: 20)

Urban Health Vulnerabilities: Understanding spatial health challenges and vulnerabilities in neighborhoods and informal settlements. (topic size: 19)

Military Tobacco Use: Examines tobacco use patterns and influences among Air Force personnel and recruits. (topic size: 16)

Smoking Cessation Pregnancy: Health providers' strategies aiding pregnant women quit smoking using tailored interventions. (topic size: 16)

Latino Health Interventions: Community health programs addressing cardiometabolic and behavioral health among older adults. (topic size: 16)

Adolescent Violence: Examines factors contributing to violent behaviors among preteens, emphasizing on alcohol use and fighting. (topic size: 16)

Perinatal Health Interventions: Evaluating digital and integrative approaches to support maternal health and infant care. (topic size: 15)

Hispanic Health Study: Examines health, environment, and social factors affecting Hispanic/Latino adults. (topic size: 15)

Social Media E-cigarette: Exploration of e-cigarette misinformation and marketing impacts on social media platforms. (topic size: 13)

Electronic Nicotine Systems: Examines usage, perceptions, and sociodemographic factors of electronic nicotine delivery system users. (topic size: 12)

Nutrition & Nursing

Behavioral Weight Loss: Focuses on interventions and strategies to promote weight loss in diverse populations. (topic size: 150)

Exercise and Health: Impact of aerobic and resistance exercises on health, fitness, and metabolic outcomes. (topic size: 109)

Child Nutrition Interventions: Examines strategies for sustaining nutrition and physical activity programs for young children. (topic size: 74)

Wearable Eating Monitoring: Devices and methods for non-invasively tracking dietary habits using wearable technology. (topic size: 72)

Postmenopausal Health: Examining weight, diet, metabolic factors impacting heart disease and diabetes post-menopause. (topic size: 54)

Children's Nutrition: Examines strategies to improve children's dietary choices in restaurants and school cafeterias. (topic size: 46)

Vibrotactile Therapy: Exploring vibration techniques to improve sensory and motor functions post-stroke. (topic size: 41)

Kinect Motion Tracking: Employing Microsoft Kinect for real-time motion tracking and assessment in healthcare and rehabilitation. (topic size: 41)

Gestational Weight Gain: Examining factors influencing weight changes during pregnancy and postpartum for intervention strategies. (topic size: 41)

Cardiac Rehabilitation: Digital interventions enhance rehabilitation, improving cardiovascular health and reducing rehospitalizations. (topic size: 40)

Workplace Food Choices: Examines health impacts of food purchases at workplace cafeterias using various studies. (topic size: 39)

Adolescent Overweight Factors: Investigates socio-economic, cultural, and genetic influences on overweight status among adolescents. (topic size: 35)

Wheelchair Energy Expenditure: Evaluating activity monitors to assess energy expenditure in manual wheelchair users. (topic size: 34)

Sarcopenic Obesity: Study of muscle loss combined with obesity and health impacts in older adults. (topic size: 31)

Upper Extremity Rehabilitation: Focuses on improving post-stroke upper extremity function using innovative therapeutic interventions. (topic size: 31)

Pedometer-Based Interventions: Strategies using pedometers to increase physical activity and manage weight effectively. (topic size: 28)

Dietary Lapses: Examining predictors of dietary lapses affecting weight management in overweight individuals. (topic size: 27)

Infant Feeding Practices: Study of breastfeeding/formula impacts and obesity prevention strategies in early childhood. (topic size: 26)

Stroke Survivor Lifestyle: Examines lifestyle factors and interventions affecting health and disability in stroke survivors. (topic size: 26)

Occupational Therapy Australia: Examines practices, challenges, and advancements in occupational therapy within the context. (topic size: 24)

Spinal Cord Injury: Exploration of costs, rehabilitation, and community integration for spinal cord injury patients. (topic size: 24)

Osteoarthritis and Obesity: Examines lifestyle and diet interventions for managing osteoarthritis in obese older adults. (topic size: 24)

Bariatric Surgery Outcomes: Examines weight loss, psychological health, and metabolic effects post-bariatric surgery. (topic size: 23)

Obesity and BMI: Examines the relationship between body mass index, obesity, and associated health risks. (topic size: 22)

Knee Exosuit Rehabilitation: Investigates knee exosuit benefits in gait assistance, rehabilitation, and muscular effort reduction. (topic size: 19)

Wearable Activity Tracking: Investigating how smartwatches track steps and link physical activity to health metrics. (topic size: 18)

Smart Gait Analysis: Advanced technologies in smart shoes optimize real-time gait monitoring and rehabilitation. (topic size: 18)

Food Insecurity: Examines effects of food insecurity on health, diet, and chronic conditions in various populations. (topic size: 16)

Healthy Aging: Exploring strategies and perspectives to enhance wellness and quality of life in older adults. (topic size: 13)

Smoking Cessation Weight Gain: Examining post-cessation weight gain challenges, especially in postmenopausal women quitting smoking. (topic size: 12)

Bluetooth-Enabled Exercise: Monitoring resistance exercise using Bluetooth devices for enhancing muscle strength and assessing usability. (topic size: 12)

Brain & Neural Science

Virtual Reality Assessment: Evaluating cognitive functions using VR technology improves accuracy and ecological validity. (topic size: 191)

Alzheimer's Disease Diagnosis: Methods and biomarkers improving Alzheimer's diagnosis and understanding cognitive decline patterns. (topic size: 112)

Adrenarche and Puberty: Investigating hormonal changes and their influence on physiology, behavior, and mental health. (topic size: 61)

Cerebral Venous Thrombosis: Research on predictors, treatment options, and complications of cerebral venous thrombosis. (topic size: 48)

Functional Brain Networks: Examines connections and dynamics of brain regions under various cognitive conditions. (topic size: 35)

Adolescent Brain Development: Examines effects of parenting on adolescent brain structures and mental health outcomes. (topic size: 19)

Gesture-Based Learning: Investigating how gesture aids in teaching complex ideas to children, enhancing understanding. (topic size: 18)

Brain Structural Asymmetry: Study of brain cortical and subcortical asymmetries linked to neurodevelopmental disorders. (topic size: 16)

P300 Speller BCI: A brain-computer interface for communication via event-related potentials in EEG responses. (topic size: 15)

Musical Coordination: Examines dynamics and patterns of coordination in group and duo musical improvisation. (topic size: 13)

ADHD Inhibition Deficits: Exploring ADHD-related cognitive deficits in inhibition, task performance, and associated brain network alterations. (topic size: 12)

Society & Business

Cookie Policy Compliance: Understanding the role of cookies in privacy and user data management on platforms. (topic size: 37)

Startup Financing Contracts: Examination of how know-how and stage-based contracts affect startup valuation and investment. (topic size: 36)

Deliberative Democracy: Evaluating public opinions and decisions through structured group discussions on social policies. (topic size: 29)

Firm Location Decisions: Examines factors influencing firms' geographical choices, including incentives, agglomeration economies, and home bias. (topic size: 29)

Foreign Direct Investment: Examines effects, policy implications, and geopolitical factors influencing foreign direct investment worldwide. (topic size: 28)

British Political Ethics: Examining ethics and conduct within politics and public perceptions of political integrity. (topic size: 28)

Latin America Politics: Examines impact of crime, finance, and reforms on the political landscape. (topic size: 27)

Irish Literary Revival: Exploration of cultural history and identity via literary and artistic works. (topic size: 24)

Financial Inclusion Disparities: Examines racial and ethnic gaps in financial service access and retirement planning. (topic size: 24)

Peer Review Acknowledgements: Expressions of gratitude towards individuals who contributed to the peer review process. (topic size: 20)

Urban Transportation Analysis: Examines urban transport patterns, emission impacts, and traveler satisfaction across diverse modes and regions. (topic size: 20)

South Carolina Economy: Analyzes economic growth, industrial shifts, and impact of development incentives. (topic size: 18)

Sustainable Hospitality Operations: Examines eco-certifications and resource efficiency's impact on hotel sustainability and performance. (topic size: 13)

Direct-to-Consumer Advertising: Examines the impact of consumer-targeted prescription drug advertising on perceptions and sales. (topic size: 12)

Medical Care Systems

Emergency Workflow: Investigating workflows in emergency settings to enhance patient care and system efficiency. (topic size: 101)

Nursing Home Quality: Evaluates nursing home quality metrics including staffing, resident satisfaction, care outcomes, and costs. (topic size: 75)

Implementation Science: Exploring frameworks to optimize healthcare practices through evidence-based implementation research strategies. (topic size: 51)

Gamified Mobile Health: Using game elements in apps to enhance engagement in digital health interventions. (topic size: 41)

Mobile Health Interventions: Implementing SMS systems to support maternal and neonatal health. (topic size: 36)

PACE Programs: Evaluates care models and outcomes for the frail elderly in all-inclusive health settings. (topic size: 32)

Nurse Practitioner Burnout: Examining burnout in nurse practitioners and its impact on primary care quality. (topic size: 31)

Emergency Trauma Care: Studying trauma treatment and epidemiological challenges in low- and middle-income regions. (topic size: 30)

Telemedicine Innovations: Advancements and challenges in telemedicine across various medical specialties post-COVID-19. (topic size: 30)

Global Emergency Medicine: Examining emergency care strategies and research priorities in various global contexts. (topic size: 30)

Trauma Center Quality: Evaluating quality and outcomes in trauma centers, focusing on mortality and complications. (topic size: 28)

Urology Workforce: Analysis of trends in demographics and gender disparities within the urology workforce. (topic size: 25)

Coronary Artery Bypass: Surgical practices, outcomes, and disparities in coronary artery bypass grafting procedures. (topic size: 23)

Pediatric Medication Dosing: Error prevention in administering liquid medications to children by improving dosing accuracy. (topic size: 23)

Health Literacy: Understanding and addressing health literacy impacts clinical outcomes in pediatric and low-income populations. (topic size: 22)

Pregnancy Monitoring: Monitoring physiological changes and stress during pregnancy using heart rate variability analysis. (topic size: 21)

Extracorporeal Membrane Oxygenation: Prolonged ECMO support impacts survival outcomes in pediatric and adult cardiac patients. (topic size: 20)

Veterans Health Practices: Evaluation of sustainment and diffusion strategies within Veterans Health Administration systems. (topic size: 17)

Remote Health Monitoring: Systems for tracking patient health remotely to optimize care and reduce costs. (topic size: 16)

Nursing Home Disparities: Examines racial and economic disparities in care quality in nursing homes. (topic size: 15)

AI Trust Healthcare: Building clinician trust in AI for effective integration in healthcare systems. (topic size: 11)
